# The clinical, economic, and humanistic burden of treatments for exocrine pancreatic insufficiency and cost-effectiveness of treatments: A systematic literature review

**DOI:** 10.1097/MD.0000000000039224

**Published:** 2024-08-16

**Authors:** Paula Chu, Jasmina Mioc, Owen Henry, Peter O’Donovan

**Affiliations:** a Organon International GmbH, Lucerne, Switzerland; b Organon Canada, Kirkland, Quebec, Canada; c Adelphi Values PROVE™, Bollington, UK

**Keywords:** burden, cost-effectiveness, epidemiology, exocrine pancreatic insufficiency, quality of life

## Abstract

**Background:**

To examine the burden of exocrine pancreatic insufficiency (EPI), specifically the clinical impact of EPI on patients, their quality of life (QoL) and the cost-effectiveness of existing treatments.

**Methods:**

A systematic literature review was conducted using key search terms for the clinical, economic, and humanistic burden. Databases were searched from 2010 to 2022, with articles screened independently by 2 reviewers at abstract and full-text stage against pre-defined eligibility criteria.

**Results:**

Seventy-one publications were identified that reported relevant clinical, humanistic, and economic data. Prevalence and incidence of EPI varied across identified studies; EPI appears to be especially prevalent as a comorbid condition in patients with cystic fibrosis. EPI has a large impact on QoL, with lower QoL scores in patients with EPI compared with those without EPI. The instruments used to assess QoL, however, were inconsistent across studies. Where reported, economic burden studies highlighted that patients with EPI have higher healthcare resource utilization compared with those without, with costs increasing with disease severity.

**Conclusion:**

This systematic literature review highlights that patients with EPI have higher treatment costs and lower QoL scores than patients without EPI. The prevalence of EPI as a comorbid condition is high, particularly in patients with cystic fibrosis.

## 1. Introduction

Exocrine pancreatic insufficiency (EPI) is a condition characterized by a deficiency of exocrine pancreatic enzymes (mainly pancreatic lipase), resulting in the inability to digest food properly, or maldigestion.^[1]^ A number of pancreatic disorders and clinical conditions can cause EPI, including but not limited to chronic pancreatitis (CP), pancreatic cancer, pancreatic surgery, and cystic fibrosis (CF), in addition to extra-pancreatic disorders such as diabetes mellitus and inflammatory bowel disease.^[1,2]^

In the current clinical landscape, the gold standard for diagnosis of EPI is to assess fat maldigestion using quantification of the coefficient of fat absorption (CFA) after 72-hour fecal fat determination.^[1]^ However, the test for CFA is burdensome and is not commonly used in clinical practice.^[1,3]^ Instead, noninvasive indirect tests for exocrine pancreatic function are often conducted, including the determination of fecal elastase-1 (FE-1) levels.^[1,3,4]^ While commonly used in clinical practice, the more practical FE-1 test comes with challenges, as it is associated with high false positive rates. This is due to the FE-1 test being contingent on EPI severity, stool consistency at time of sampling, and other medical conditions.^[5]^

Diagnosis and treatment is crucial to prevent clinical manifestations of EPI, including steatorrhea, weight loss, severe malnutrition, and maldigestion, which can negatively impact patients’ quality of life (QoL) and significantly impair patients’ daily function.^[1]^ Due to the aforementioned diagnostic difficulties, estimating the prevalence of EPI can be challenging. This is further complicated by the fact that EPI exhibits similar symptoms to other common diseases and is often not tested for, especially in the early stages.^[6]^ Patients with EPI are at an increased risk of mortality, with EPI reported as a significant independent risk factor for mortality in patients with CP.^[4,7]^

The standard management of EPI includes Pancreatic Enzyme Replacement Therapy (PERT), such as Creon® (pancreatin mini-microspheres [MMS]) and Zenpep®, along with a standard high calorie intake nutrition (oral or tube-feeding).^[8]^ The optimal dosing of PERT remains unclear, since it can be impacted by patients’ residual pancreatic function and the fat content in their diet. As EPI is a chronic condition, patients may require continued treatment with PERT for the rest of their life, which may result in substantial economic burden.^[5]^ However, the true economic burden of PERT is currently unknown, with few cost-effectiveness analyses published in literature.

There has been a multitude of recent studies investigating the clinical, humanistic, and economic burden of EPI and, therefore, an updated systematic literature review (SLR) was necessary to collate the latest evidence. This SLR aimed to examine the burden of EPI, specifically the clinical impact on patients, their QoL, and the cost-effectiveness of existing treatments.

## 2. Materials and methods

This SLR was conducted in line with the Preferred Reporting Items for Systematic Reviews and Meta-Analyses (PRISMA) guidelines^[9]^ and the Cochrane Collaboration guidance.^[10]^ Relevant articles were identified using the following electronic databases: Embase^®^, Medline^®^, Evidence-Based Medicine Reviews, PsycInfo and Econlit via the Ovid search platform. The searches were designed to capture publications relating to the burden of EPI (including the clinical, humanistic, and economic burden) and cost-effectiveness of treatments for EPI. The SLR included a 12-year time horizon (2010–2022) to ensure publications captured were recently published and of high relevance. In addition to the database searches, a gray literature review and a review of conference proceedings (Digestive Disease Week, The Professional Society for Health Economics and Outcomes Research, The European Society of Neurogastroenterology and Motility, American Pancreatic Association, European Pancreatic Club) from 2021 to September 26th, 2022 was conducted to identify evidence not reported in the published literature. The search strategies used to conduct the SLR are provided in Tables S1–S8, Supplemental Digital Content, http://links.lww.com/MD/N341

Two independent reviewers screened titles, abstracts, and full-text publications, with a consensus reached over any disagreements by a third reviewer. The full Population, Intervention, Comparison, Outcome, Time, and Study design criteria are provided in the supplementary appendix (Tables S9 and S10, Supplemental Digital Content, http://links.lww.com/MD/N341). Key criteria for inclusion were patients with EPI (attributed to a number of medically defined pancreatic diseases or any other medically defined pancreatic disease that might require PERT) and studies reporting clinical, humanistic, and economic burden outcomes. Additionally, only records published in English were included. All relevant data were extracted from the included publications by a single reviewer and a full quality check was subsequently conducted by a senior reviewer.

Included records were also subjected to a quality assessment to determine the strength and applicability of their findings. The critical appraisal tools published in the Joanna Briggs Institute Manual for Evidence Synthesis were used.^[11]^

## 3. Results

A total of 71 publications (Figure S1, Supplemental Digital Content, http://links.lww.com/MD/N341) relating to the burden of disease were identified, of which 63 studies reported on the clinical burden of EPI, 11 reported on the humanistic burden, and 9 reported on the economic burden of EPI.

Of the 63 included studies reporting on the clinical burden, there were 62 observational studies and one survey. The majority of identified clinical burden studies were conducted in the United States (n = 10),^[12–21]^ Spain (n = 6),^[7,22–26]^ and India (n = 5)^[27–31]^ across a data collection period ranging from 3 months to 28 years. Eleven humanistic studies were identified and included, with the data collection period ranging from 2 months to 10 years and conducted across 9 countries.^[13,25,32–38]^ Nine studies also reported on economic burden of EPI; however, only 3 reported on a population that consisted solely of patients with EPI.^[26,38–43]^ The remaining 4 studies included mixed populations, although all included a large proportion of patients with EPI. These publications were included in the SLR due to the low number of studies. Studies reporting economic burden data were conducted across 7 different countries and collected over a period ranging from 5 months to 19 years. The characteristics of these included studies are presented in Table S11, Supplemental Digital Content, http://links.lww.com/MD/N341.

This SLR identified patients with EPI in a range of population groups, such as those with CF, acute pancreatitis (AP), and following pancreatic surgery. The majority of publications reported on patients with CP (n = 18) and diabetes mellitus (n = 9). The mean age of patients was reported in 47 studies, ranging from 7.5 years in patients with CF to 67 years in patients who underwent total pancreatectomy. The definition and detection method for diagnosing EPI within included studies varied. The FE-1 test was the most frequently used, with less than 200 μg/g considered EPI, but some studies stratified EPI further into moderate or severe classifications. Additionally, other methods of detection were used, such as the 13-C mixed triglyceride breath test.

### 3.1. Clinical burden of EPI

The prevalence of EPI was recorded across 55 publications (Table S12, Supplemental Digital Content, http://links.lww.com/MD/N341). Of the studies that reported prevalence of EPI as a comorbid condition to other diseases, such as CP and CF, the prevalence of EPI varied and was typically reported on small sample populations. For example, in a control group of patients in Serbia, prevalence was 2.5%.^[44]^ In contrast, a study in a small sample (n = 14) of patients with CF and EPI on the island of São Miguel, Portugal, a prevalence of 100% was reported and included.^[45]^ The study with the largest sample size was Domínguez Muñoz et al, which included 1900,751 patients with CP treated in Spain, and reported a prevalence of EPI of 38.8%.^[23]^ In patients with CP, the prevalence of EPI ranged from 13% (n = 89)^[15]^ to 82.2% (n = 60).^[20]^

Similarly, the incidence of EPI varied across the 9 studies reporting these data and the sample sizes were small. The lowest recorded incidence by Neophytou et al was 2% in patients undergoing enucleation for benign pancreatic neoplasm in France (n = 92).^[46]^ On the other hand, Kroon et al reported an incidence of 88% in patients following pancreatoduodenectomy in the Netherlands (n = 152), highlighting the clinical burden of EPI in this population.^[47]^ Two publications reported the incidence of EPI within CP populations, closely ranging from 31.3% (n = 52, unknown country)^[48]^ to 36.23% (n = 1717, United States).^[16]^

The availability of mortality data was limited. One publication that collected follow-up data for patients with CP in Spain reported that 20.6% of patients with CP and EPI died during follow-up (n = 126).^[7]^

These results display the variability of prevalence and incidence of EPI. Nonetheless, the largest study investigating prevalence suggests a significant clinical burden associated with EPI. EPI is often reported as a comorbid condition with the highest prevalence reported in patients with CF.

### 3.2. Humanistic burden of EPI

The studies investigating humanistic burden of EPI used QoL instruments and/or patient-reported outcomes (PRO) questionnaires to demonstrate the impact of EPI and its symptoms on patients’ QoL (Table 1 and Table S13, Supplemental Digital Content, http://links.lww.com/MD/N341). We found a lack of consistency in the QoL assessment instrument used, with 4 out of 8 studies using the European Organization for Research and Treatment of Cancer quality of life questionnaire-C30,^[25,34,35,38]^ one study using the gastrointestinal quality of life index,^[33]^ and 2 studies using the 36-item Short-Form Health Survey (SF-36).^[13,37]^

**Table 1:** Summary of humanistic burden studies.

Author, year	Study design	Comorbidities	Sample size	Population demographics	Diagnostic/detection method	Key findings
			n			
Lamarca A. 2021^[36]^	Prospective observational analysis	Patients with acute pancreatic cancer	75	NR	1. Full dietetic assessment (including Mid-Upper Arm Circumference, handgrip and stair climb test)	Presence of EPI related symptoms: flatulence (84.0%), weight loss (84.0%), abdominal discomfort (50.0%), steatorrhea (48.0%).
					2.Full nutritional blood panel	
					3.FE-1 levels	
					4.C mixed triglyceride breath test (for the diagnostic cohort)	
Latenstein A.E.J. 2021^[38]^	Multicenter observational data-base analysis	Patients with a history of pancreas surgery due to premalignant or benign disease	153	Median (IQR) age at operation: 63.0 years (54.0–70.0)	NR	The VAS (EQ-5D-5L) was 76 (±17) versus 82 (±0.4) in the general population (*P* < .001).
				Gender: Female 66 (43.0%), Male 87 (57.0%)		The mean global health status score (EORTC QLQ) was 78 (±17) versus 78 (±17) in the general population, (*P* = 1.000).
						Fatigue, insomnia, and diarrhea were clinically relevantly worse in patients.
						New-onset diabetes mellitus was present in 22 patients (14%).
Oh M.Y. 2021^[34]^	Prospective single-center observational analysis	Patients who underwent pancreatic surgery (elective single stage total pancreatectomy)	30	Mean (SD) age at baseline: 64.3 years (±12.32)	NR	The global health status score (EORTC QLQ) showed no significant difference over time following surgery.
				Gender: Female 17 (56.7%), Male 13 (43.3%)		Bowel movement frequency increased over time and relative body weight was lowest after 1 year post-surgery.
						At 3 months post-surgery, on the EORTC QLQ physical and role function scores as well as symptoms of fatigue, constipation, and digestive difficulties worsened significantly.
Raun A.M.T. 2021^[49]^	Open label randomized controlled cross-over trial	Cystic fibrosis	30	Mean (SD) age at baseline: 7.5 years (±5.7)	NR	The most frequent symptoms reported were flatulence and foul-smelling stools in 42.9% (n = 12/28), lack of appetite in 35.7% (n = 10/28), and abdominal pain in 29.4% (n = 5/17).
				Gender: Female 17 (56.7%), Male 13 (43.3%)		Most patients reported “no problem” to all GI-related QoL questions (GIQLI) at baseline (n = 13–16/17, 76.5–94.1%).
Kempeneers M.A. 2020^[37]^	Prospective observational data-base analysis	Chronic pancreatitis	304	Mean (SD) age at baseline: 58.0 years (±11.0)	1.No symptoms of steatorrhea	Patients with definite EPI (n = 304) had significantly more malabsorption symptoms, a lower body mass index, and aberrant defecation.
				Gender: Female 101 (33.2%), Male 203 (66.8%)	2.No pancreatic enzyme replacement therapy	Lowered quality of life was not independently associated with EPI. However, on the physical component of the QoL SF-36 questionnaire, the score was significantly lower in patients with PEI compared with patients without PEI (40.5 vs 44.0; *P* < .001).
					Both without a documented abnormal exocrine function test or with a documented normal exocrine function test.	Of the EPI patients using PERT, 47% still reported steatorrhea.
Stoop T.F. 2020^[32]^	Prospective single-center observational analysis	Patients who underwent pancreatic surgery (elective single stage total pancreatectomy)	53	Median (range) age at baseline: 67.0 years (18.0–83.0)	PEI was assessed using a questionnaire which included items such as the Bristol Stool Form Scale	Overall QoL was reduced compared with a general population (66.7% vs 76.4%) but did not differ with preoperative outcomes (n = 39, 66.7% vs 66.7%; *P* = .553) according to the EORTC QLQ-C30.
				Gender: Female 27 (50.9%), Male 26 (49.1%)		The PAID20 revealed emotional burnout in 7 patients (13.2%).
						Overall, 27 patients (50.9%) reported to have steatorrhea during a median of 2 days (IQR 0–4) in the past week.
Smith Z.L. 2019^[13]^	Cross-sectional single-center observational analysis	Walled-off pancreatic necrosis	14	Mean (SD) age baseline: 52.0 years (±15.0)	Clinical steatorrhea or imaging suggestive of chronic calcific pancreatitis with PERT use, abnormal FE-1 levels, abnormal secretin-enhanced pancreatic function testing or abnormal quantitative stool fat collection on 100 g/24 hour diet (>7 g/24 h).	Development of exocrine insufficiency was predictive of lower total SF-36 scores (*P* = .016). In patients developing exocrine insufficiency versus healthy controls, poorer scores in the physical role (*P* < .001), general health (*P* < .001), vitality (*P* = .001), and emotional role (*P* = .029) domains were observed.
				Gender: Female 9 (64.3%), Male 5 (35.7%)		One-year all-cause mortality was 6.2%, and disease-related mortality was 3.7%.
Marra-Lopez Valenciano C. 2018^[25]^	Exploratory, qualitative, concept-elicitation interview analysis	Chronic pancreatitis	41	Mean (SD) age at baseline: 58.6 years (±9.0)	FE-1 levels in stool samples.	Regarding nutritional status, the following differences were observed (EPI vs Non-EPI): BMI (23.9 vs 25.7 kg/m^2^; *P* = .03), Vitamin A (0.44 vs 0.53 mg/L; *P* = .048) and Vitamin E (11.2 vs 14.4 μg/mL, *P* = .03).
				Gender: Female 5 (12.2%), Male 36 (87.8%)		EPI patients showed a worse EORTC QLQ score on physical (93.3 vs 100; *P* = .048) function.
Johnson C.D. 2017^[50]^	Observational primary research interview analysis	Chronic pancreatitis or cystic fibrosis	61	Mean age at baseline: 36.0 years	Physician confirmed diagnosis of PEI.	There were 6 symptoms that were reported as the most common among the participants: pain (n = 49 [80%]), bloating (n = 39 [64%]), diarrhea (n = 46 [76%]), nausea/vomiting (n = 39 [64%]), weight loss (n = 41 [67%]), and tiredness/fatigue (n = 25 [41%]).
				Gender: Female 29 (48.0%), Male 32 (52.0%)		The most common daily activities impacted were “housework,” “holidays,” and “traveling” (all n = 6 [10%]).
D’Haese J.G. 2014^[33]^	Prospective multicenter observational analysis	Chronic pancreatitis	294	Mean (SD) age at baseline: 62.7 years (±12.6)	NR	Following initiation of treatment, the proportion of patients experiencing gastrointestinal symptoms and recurrent pain after 1 year was significantly reduced (*P* < .001). The alleviation of symptoms was reflected in GIQLI score improvements at 1 year (*P* < .001),
				Gender: Female 132 (44.9%), Male 162 (55.1%)		
Halloran C.M. 2011^[35]^	Prospective single-center observational analysis	Patients who underwent pancreatectomy for neoplasia	40	Median (IQR) age at baseline: 65.0 years (54.5–70.3)	FE-1 levels < 200 μg/g	Overall, QoL increased at 6 (*P* = .0212) and 12 (*P* < .0001) months after surgery.
				Gender: Female 18 (45.0%), Male 22 (55.0%)		EPI did not affect BMI, QoL, and symptoms in patients undergoing surgery.

BMI = body mass index, EORTC QLQ = European Organisation for Research and Treatment of Cancer Quality of Life Questionnaire, EPI = exocrine pancreatic insufficiency, EQ-5D-5L = EuroQol-5-Dimension-5-Likert, GIQLI = Gastrointestinal Quality of Life Index, IQR = interquartile range, NR = not reported, PAID20 = Problem Areas in Diabetes scale, PERT = pancreatic enzyme replacement therapy, QoL = quality of life, SD = standard deviation, SF-36 = 36-item Short-Form Questionnaire, VAS = Visual Analogue Scale.

In 4 studies investigating the impact of EPI symptoms on patients, weight loss resulting from malabsorption of nutrients and maldigestion was consistently reported.^[33,37,38,50]^ These studies also found that weight loss was mitigated with PERT, highlighting the effectiveness of PERTs in increasing nutrient absorption.^[33,37,38,50]^ Kempeneers et al investigated symptoms in patients with CP and EPI and reported that 36% of patients receiving PERT experienced unintentional weight loss compared with 55.3% in patients not receiving PERT (n = 987, *P* = .022).^[37]^ The trend of symptom improvement following PERT therapy was further observed across 7 studies using performance-related outcomes to assess the impact of therapy on EPI symptoms. Kempeneers et al also reported on the QoL of patients with CP and EPI and patients with CP without EPI using the SF-36.^[37]^ Both the physical and the mental components scores were lower in patients with EPI (40.5 and 45.5, respectively) than in patients without EPI (44 and 46.3, respectively) (n = 987).^[37]^ Additionally, QoL scores improved following EPI treatment.

Together, these data show that EPI has a large impact on patient QoL as indicated by the lower QoL scores in patients with EPI compared with patients without EPI. Further, use of EPI treatments, such as PERT, is associated with an improvement of EPI symptoms and QoL scores.

### 3.3. Economic burden of EPI

The economic burden of EPI was recorded in 8 studies, but only 3 reported exclusively in EPI patients (Table 2 and Table S14, Supplemental Digital Content, http://links.lww.com/MD/N341). The remaining studies focused on the economic burden of other diseases, such as CP and CF. Given the paucity of economic burden data, they were subsequently included in this SLR. Of the 3 publications in EPI patients only, a trend of higher costs for treatment in the EPI patient populations was observed.^[26,42,43]^ Gupta et al investigated the direct costs associated with EPI in 2021 and reported that the average retail cost of 5 branded PERTs for EPI in the United States was estimated at 1288–1860 USD for a 15-day supply.^[42]^ Additionally, Eidt-Koch et al provided a comparison of mean costs of treatment for patients with CF with or without EPI in Germany in 2006 and found that the mean cost was 23,305 EUR for patients with EPI and 16,284 EUR for patients without EPI.^[43]^ Eid-Koch et al further investigated treatment costs for patients with CF, reporting that the price of 1 unit of oral pancreatin at the lowest dose (Creon® 10,000) was 0.24 EUR and the daily treatment costs for EPI at 0.24–8.67 EUR, demonstrating the extra cost associated with EPI in related disease populations.^[43]^ Whilst these results are not EPI specific, 82.4% of the patients with CF had EPI and therefore received treatments such as pancreatin.

**Table 2:** Summary of economic burden studies.

Author, Year	Study design	Comorbidities	Sample size	Population demographics	Key findings
			n		
Cartelle A.L. 2022^[20]^	Retrospective single-center observational analysis	Chronic pancreatitis (disabled versus non-disabled)	404 (82% have EPI)	NR	Disabled patients also had higher rates of ED visits for opioid pain medication (16.7% vs 4.2%) and higher average number of flares requiring hospitalizations (4.1 vs 1.9).
Fang K. 2022^[39]^	Retrospective single-center observational analysis	Pancreatectomy for pancreatic tumors	101 (6.9% have EPI)	Median (range) age at baseline: 45.3 years (10.0–75.0)	Mean length of hospital stay was 11.6 days (±8.1). Re-operation rate was 1.0%.
				Gender: Female 74 (73.3%), Male 27 (26.7%)	
Gupta A. 2022^[42]^	Cross-sectional descriptive analysis	NR	NR – results are specific to EPI	NR – No patients were included; the study is analyzing costs of EPI treatments	For a 15-day supply of PERTs, average retail prices ranged from $1288 USD to $1860 USD.
					For cash paying patients with coupons, the lowest price for these drugs ranged from $1072 USD to $1514 USD.
Latenstein A.E.J. 2021^[38]^	Multicenter observational analysis	Patients with a history of pancreas surgery	153 (64% have EPI)	Median (IQR) age at operation: 63.0 years (54.0–70.0)	Median (IQR) length of hospital stay: 11 days^[7–21]^
				Gender: Female 66 (43.0%), Male 87 (57.0%)	
Pranger B.K. 2021^[40]^	Retrospective multicenter observational analysis	Patients following pancreatic resection	230 (19.6% have EPI)	Median (IQR) age at operation: 32.0 years (25.0–37.0)	Median (IQR) length of hospital stay: 11 days.^[9–17]^
				Gender: Female 143 (62.2%), Male 87 (37.8%)	Median (IQR) procedure time: 300 minutes (250–430).
Bartholdy A. 2020^[41]^	Retrospective single-center observational analysis	Walled-off necrosis	125 (22% have EPI)	Median (range) age at baseline: 57.0 years (21.0–80.0)	Readmission rate: 45 (36%) patients had one or more WON-related readmissions, and 50 (40%) patients had one or more non-WON-related readmissions.
				Gender: Female 57 (45.6%), Male 68 (54.4%)	Twenty-two (18%) patients were on PERT at discharge. This number remained unchanged during FU.
Dieguez-Castillo C. 2020^[26]^	Cross-sectional single-center observational analysis	Chronic pancreatitis	30 (100% have EPI)	Mean age at baseline: 55.8 years	Number of patients requiring PERTs: 21 (70%).
				Gender: Female 2 (6.7%), Male 28 (93.3%)	Number of patients requiring surgical treatment: 10 (20%).
Eidt-Koch D. 2010^[43]^	Multicenter cross-sectional observational analysis	Cystic fibrosis	301 (82.4% have EPI)	Mean age: 19.9 years	Treatment costs were higher in patients with EPI compared to those without EPI: 23,305 EUR (EPI patients) versus 16,284 EUR (non-EPI patients).
				Gender: NR	Mean daily medication costs per day for selected medications (EUR): Tobi® (2.82–118.33); Colistin® (8.73–69.86); Pulmozyme® (16.59–33.18); Gernebcin® (2.84–37.93); Kreon 10,000® (0.24–8.67); Kreon 25,000® (0.49–23.71); Kreon 40,000® (1.81–25.30); Sempera® (3.15–12.60); Zithromax® (0.66–18.47); Viani® (2.22–6.95)

ED = emergency department, EPI = exocrine pancreatic insufficiency, EUR = Euro, IQR = interquartile range, NR = not reported, PERT = pancreatic enzyme replacement therapy, SD = standard deviation, USD = United States Dollar, WON = walled-off necrosis.

Four studies reported healthcare resource utilization (HCRU) data;^[20,26,39,40]^ however, only Dieguez-Castillo et al reported HCRU data for a population solely focusing on patients with EPI from 2015 to 2016. In this study, EPI contributed to an increased overall resource use in Spain and as expected, HCRU increased with EPI severity.^[26]^ For example, 21 (70%) patients with CP and EPI required PERT treatment, with 2 of these 21 patients having mild/moderate EPI compared to 19 with severe EPI. Ten (33.3%) of the patients with CP and EPI required surgical treatment, with 3 of these classified as mild/moderate EPI and 7 classified as severe EPI patients.^[26]^ Cartelle et al also reported HCRU data for a cohort of disabled and non-disabled CP patients from 2016 to 2021.^[20]^ A diagnosis of EPI was significantly associated with disability (OR 3.53, 95% CI 1.91–6.97), with 82% of the disabled patients having EPI compared to 55.5% of the non-disabled patients. The number of hospitalizations due to flare ups was similar for both disabled and non-disabled patients (4% and 3.2%, respectively). However, resource use was greater for disabled patients, with 34.7% requiring celiac blocks (a treatment which blocks the celiac plexus nerves from sending pain signals to the brain) compared to 9.8% of non-disabled patients and higher rates of emergency department visits for opioid pain medication in disabled (16.7%) compared with non-disabled patients (4.6%).^[20]^

The SLR identified one economic evaluation: a cost-effectiveness model performed from a Polish payer perspective (Table 3 and Figure S2, Supplemental Digital Content, http://links.lww.com/MD/N341).^[6]^ The study evaluated the cost-effectiveness of using Creon® to treat EPI.^[6]^ No PERT treatment was used as a comparator due to the limited data on alternative treatments for EPI prescribed in Poland.^[6]^ At the beginning of the model all patients were considered to be in the uncontrolled EPI state (CFA less than or equal to 80%) and either received treatment with pancreatin MMS oral capsules or no PERT treatment.^[6]^ The initial pancreatin MMS regimen used in the model was 40,000 lipase units per main meal (3-times per day) and 25,000 lipase units per snack (2-times per day).^[6]^ After a 2-week period (the initial model cycle), patients either become controlled (CFA greater than 80%) or do not improve and remain uncontrolled.^[6]^ The resulting incremental cost-effectiveness ratio in the base case of 6,312 EUR was lower than the willingness-to-pay threshold of 24,148 EUR in Poland at the time of reporting (March 2012); thus, pancreatin MMS was considered as a cost-effective treatment for EPI from a Polish payer perspective.^[6]^ The identification of only one published economic evaluation highlights that further research is required into the cost-effectiveness and budget impact of EPI treatments from different country payer perspectives.

**Table 3:** Summary of economic models and utilities outcomes.

Author, Year	Model design	Country Intervention Currency year Currency	Time Horizon	Total costs	QALYs	ICER (cost per QALY)
Morawski J.H. 2012^[6]^	Markov model – 3 health states (uncontrolled EPI, controlled EPI, and death) from a payer perspective	Poland Pancreatin MMS 2011 EUR	20 years	8223	9.45	6312
			10 years (sensitivity analysis)	5816	6.36	6666
			5 years (sensitivity analysis)	3473	3.64	7036

EUR = Euro, ICER = incremental cost-effectiveness ratio, LY = life year, MMS = mini-microspheres, NR = not reported, QALY = quality-adjusted life year.

Overall, these results suggest that the economic burden of EPI is high, with higher associated treatment costs for patients with EPI compared with patients without EPI and increased costs with increased severity.

## 4. Discussion

This SLR highlights the sizeable burden associated with EPI across multiple comorbid conditions. Despite variability across studies, EPI had a high prevalence and incidence. The studies identified in this SLR assessed the clinical burden of EPI through a multitude of outcomes across a broad range of patients with or without other diseases (such as CF or CP) from pediatric to older adults. The humanistic burden of EPI was evident as indicated by PRO and QoL measures, with EPI consistently associated with lower scores (indicating lower QoL). Additionally, the economic burden studies report an association between EPI and higher treatment costs and HCRU.

This SLR identified a large evidence base of publications investigating the clinical burden of EPI, covering a diverse range of populations with data collected across multiple countries and regions. Due to the small study sizes in publications reporting EPI prevalence and incidence, it is unlikely that the data reported in this SLR are fully representative of the true prevalence and incidence of EPI. Additionally, high variability in incidence and prevalence data was observed, possibly due to the different clinical guidelines on diagnosis and classification of EPI in the included countries. Further, in the majority of publications that reported prevalence and incidence data, EPI was identified as a comorbid condition and as such was not the focus of the studies. Despite this, collectively the publications identified within this SLR suggest that EPI is most prevalent and most severe in patients with CF. The largest study that investigated EPI prevalence indicates that the clinical burden of EPI may be greater than currently predicted in published literature.^[23]^ However, there is a need for further, larger, population-based studies, with a focus solely on patients with EPI to be able to reliably estimate the burden of EPI in the different clinical populations. Our findings are in line with recent review articles that indicate that the prevalence of EPI varies depending on the underlying disease (with most patients with CF developing EPI) and varies across studies within the same disease.^[3,5]^

With regard to the humanistic burden of EPI, the limited number of included studies means only limited conclusions can be drawn. The instruments used to assess QoL varied across the studies, highlighting a clear data gap to be investigated in future publications. The humanistic burden of EPI was high, with lower QoL scores observed for patients with EPI compared with those without. Further, studies reported that patient QoL scores improved when treated with EPI therapy such as PERT due to improved symptoms (e.g., slowing or stopping weight loss) when compared to those who did not receive EPI treatment. However, this SLR identified that the QoL instruments and the PROs used were not consistent or specific to the severity of symptoms experienced by patients with EPI. As EPI is subjective, there is a need for standardized, validated, and EPI-specific PRO measures to be used consistently in future research to accurately reflect patients’ QoL. For example, the use of the Pancreatic Exocrine Insufficiency Questionnaire (PEI-Q), which is reported in published literature to be both valid and reliable, could be used.^[51]^

Of the 9 studies reporting the economic burden of EPI, only 3 studies reported results exclusively to EPI patients.^[26,42,43]^ Treatment costs were greater for patients with EPI than those without EPI and HCRU was greater in patients with more severe disease. Like the identified clinical burden publications, a minority of publications reported data for patients with EPI only, with the majority typically reporting EPI as a comorbid condition in another population group. Consequently, data on costs specific to patients with EPI was largely unavailable. The results presented in this SLR suggest there is a cost burden associated with EPI, but a gap exists in the evidence. Additional research could further investigate the economic burden of EPI.

Risk of bias was assessed across all peer-reviewed studies using a validated quality assessment tool. The overall risk of bias across included studies was deemed to be low. Of the studies included, 38 were deemed to have a low risk of bias, 5 were deemed to have a potentially high risk of bias (mainly due to a lack of reporting on methods) and 6 were unclear.

To our knowledge this is the first SLR that explores the burden of EPI as well as the economic evaluation of treatments for EPI. The methods used to conduct the SLR were robust, with recognized methodologies followed and comprehensive database and gray literature searches conducted. Additionally, the SLR had a number of limitations, including a lack of published literature for a number of aspects of the review, specifically for the economic and humanistic burden of EPI. In relation to the economic burden, 5 studies were included that were not fully comprised of patients with EPI, with only 3 of 8 studies focusing specifically on the population of interest.^[26,42,43]^ These 5 studies were included to address data gaps, meaning these data may not be completely applicable to the EPI population. However, it should be noted that the majority of these studies had a high inclusion rate of patients with EPI and therefore would likely be representative and relevant. Given the high prevalence of the disease, future studies should solely look at the economic burden of patients with EPI as this SLR only identified one cost-effectiveness model.

Overall, this SLR highlights the significant burden on patients associated with EPI. The review has identified a number of evidence gaps including a scarcity of economic, humanistic, and cost-effectiveness data which should be addressed in the future to fully understand the burden on patients. Understanding the burden of EPI is necessary to heighten the awareness of EPI both clinically and economically and further encourage discourse among clinical experts to address challenges regarding diagnosis and inconsistencies in clinical guidelines.

## Acknowledgments

The authors would like to thank and acknowledge Louise Heron, Ana Costa and James Cochrane from Adelphi Values PROVE™ for their support in conducting the literature review and drafting and revising content.

## Author contributions

**Conceptualization:** Paula Chu, Peter O’Donovan.

**Data curation:** Paula Chu, Owen Henry, Peter O’Donovan.

**Formal analysis:** Paula Chu, Owen Henry, Peter O’Donovan.

**Methodology:** Paula Chu, Peter O’Donovan.

**Project administration:** Peter O’Donovan.

**Writing – original draft:** Paula Chu, Jasmina Mioc, Owen Henry, Peter O’Donovan.

**Writing – review & editing:** Paula Chu, Jasmina Mioc, Owen Henry, Peter O’Donovan.

## Supplementary Material


