# Supplementary material for: The clinical, economic, and humanistic burden of treatments for exocrine pancreatic insufficiency and cost-effectiveness of treatments: A systematic literature review
Source: Medicine (Baltimore). 2024 Aug 16;103(33):e39224. doi: 10.1097/MD.0000000000039224 (PMC11332733; doi:10.1097/MD.0000000000039224)
Supplement: Supplementary file 1 [file medi-103-e39224-s001.pdf]

Figure 1. PRISMA diagram for burden of disease

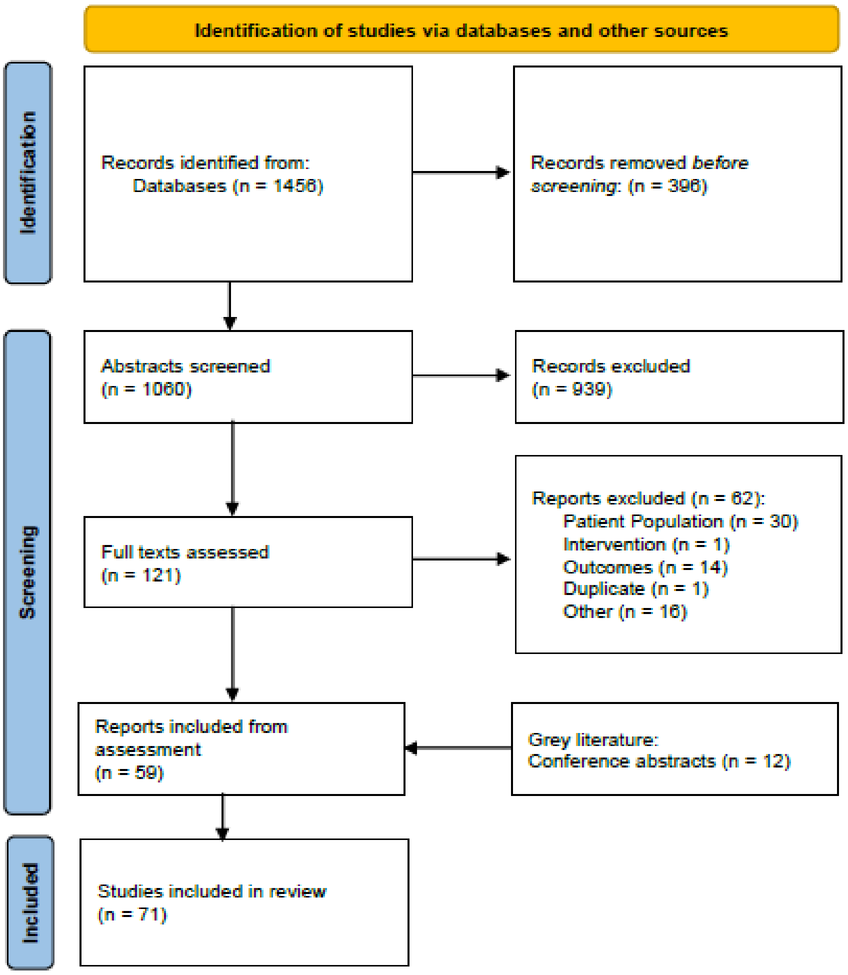

Figure 2. PRISMA diagram for economic models

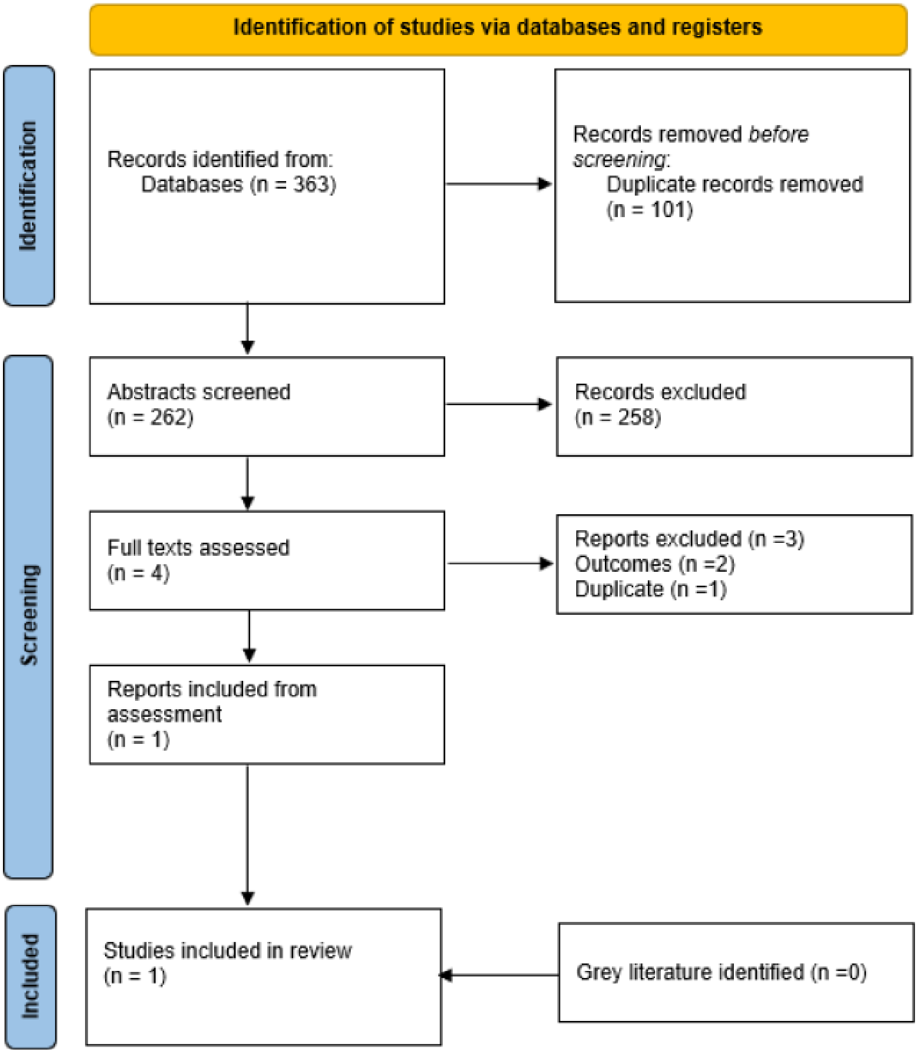

Supplementary Table 1. Burden of Disease SLR Embase Search Strategy

No	Category	Search term	Hits
1	***Population terms***	exp exocrine pancreas insufficiency/	1553
2		((pancrea* adj5 (exocrine or enzyme?) adj3 (insufficien* or deficien* or disorder? or lack* or failure? or dysfunction* or inadequa* or adequa*)) or pert).tw.	5079
3		1 or 2	5527
4	***Economic burden***	health economics/	27566
5		cost of illness/	20719
6		cost control/	69583
7		disease burden/	36619
8		medical leave/	8063
9		healthcare financing/	13738
10		medical fee/	13439
11		work disability/	5408
12		absenteeism/	17171
13		presenteeism/	2029
14		productivity/	43371
15		caregiver/	102350
16		length of stay/	241407
17		intensive care/	132103
18		hospitalization cost/	9007
19		health care utilization/	85592
20		((economic or societ* or socioeconomic or socio economic or illness or disease or patient* or caregiver* or carer*) adj3 (burden or impact or consequence?)).tw.	244146
21		(productivity or cost* or price* or pricing or pharmacoeconomic* or pharmaco-economic* or expenditure* or expens* or financ* or visit* or (length adj5 stay)).tw.	1819872
22		(resource* adj2 ("use" or usage or utili* or allocat*)).tw.	72677
23		(leave adj2 (medical or sick or disability)).tw.	7680
24		or/4-23	2438714
25		3 and 24	577
26	***Humanistic burden***	quality of life/	568139
27		quality of life assessment/	13219
28		patient-reported outcome/	43228
29		patient preference/	23970
30		psychological well being/	26417
31		caregiver burden/	9665
32		(qol or hrqol or quality of life).tw.	546941
33		((patient* or caregiver* or carer* or parent*) adj3 (satisf* or perspective* or view* or prefer* or attitude* or outcome* or burden or stress* or well being or perce* or pressur* or anxi* or depress*)).tw.	1101409
34		or/26-33	1732425

No	Category	Search term	Hits
35		3 and 34	725
36	***Epidemiological***	epidemiology/	150138
37		incidence/	513565
38		prevalence/	866429
39		mortality/	813947
40		morbidity/	379651
41		(epidemiolog* or incidence* or prevalen* or mortalit* or morbid*).tw.	3858757
42		or/36-41	4395858
43		3 and 42	1516
44		clinical study/	111019
45		case control study/	192369
46		family study/	25218
47		longitudinal study/	176089
48		retrospective study/	1300321
49		prospective study/	789928
50		cohort analysis/	892799
51		(cohort adj (study or studies)).mp.	418935
52		(case control adj (study or studies)).mp.	251886
53		(follow up adj (study or studies)).mp.	66571
54		(observational adj (study or studies)).mp.	351862
55		(epidemiologic* adj (study or studies)).mp.	115865
56		(cross sectional adj (study or studies)).mp.	551208
57		((real word adj (study or studies or evidence)) or RWE).mp.	1420
58		or/44-57	3700574
59		43 and 58	594
60	***Total before any exclusion and limits***	25 or 35 or 59	1516
61		exp case study/ or exp case report/ or exp letter/ or exp editorial/ or exp preliminary communication/ or exp erratum/ or exp note/	4967165
62		(exp animal/ or exp invertebrate/ or nonhuman/ or animal experiment/ or animal tissue/ or animal model/ or exp plant/ or exp fungus/) not (exp human/ or human tissue/)	6105504

No	Category	Search term	Hits
63	***Total after exclusion terms***	60 not (61 or 62)	1361
64	***Total after time***	limit 63 to yr="2010 -Current"	1230
65	***Total after limiting to conference from the past 2/3 years***	exp conference paper/ or conference abstract/ or (conference adj (abstract or paper or review or proceeding)).pt.	5328048
66		limit 65 to yr="2010 - 2019"	3, 066301
67		64 not 66	795
68		limit 67 to english language	771

Supplementary Table 2. Burden of Disease SLR Medline search strategy

No	Category	Search term	hits
1	***Population terms***	exp Exocrine Pancreatic Insufficiency/	2364
2		((pancrea* adj5 (exocrine or enzyme?) adj3 (insufficien* or deficien* or disorder? or lack* or failure? or dysfunction* or inadequa* or adequa*)) or pert).tw.	3312
3		1 or 2	4537
4	***Economic burden***	cost of illness/	30940
5		health care costs/	43496
6		"health care economics and organizations"/	0
7		"Cost Control"/	21652
8		Sick Leave/	6587
9		Healthcare Financing/	1210
10		Fees, Medical/	5510
11		Absenteeism/	9671
12		Presenteeism/	545
13		Efficiency/	15104
14		Caregivers/	46994
15		"Length of Stay"/	100377
16		Critical Care/	58834
17		Hospital Costs/	11871
18		"Patient Acceptance of Health Care"/	53785
19		((economic or societ* or socioeconomic or socio economic or illness or disease or patient* or caregiver* or carer*) adj3 (burden or impact or consequence?)).tw.	155983
20		(productivity or cost* or price* or pricing or pharmacoeconomic* or pharmaco-economic* or expenditure* or expens* or financ* or visit* or (length adj5 stay)).tw.	1356970
21		(resource* adj2 ("use" or usage or utili* or allocat*)).tw.	49862
22		(leave adj2 (medical or sick or disability)).tw.	6237
23		or/4-22	1721296
24		3 and 23	249
25	***Humanistic burden***	"Quality of Life"/	249498
26		patient-reported outcome measures/	12188
27		stress, psychological/	131722
28		Patient Preference/	10402
29		Caregiver Burden/	449
30		(qol or hrqol or quality of life).tw.	346045
31		((patient* or caregiver* or carer* or parent*) adj3 (satisf* or perspective* or view* or prefer* or attitude* or outcome* or burden or stress* or well being or perce* or pressur* or anxi* or depress*)).tw.	719544
32		or/25-31	1183998
33		3 and 32	334
34	***Epidemiology***	Epidemiology/	12559
35		Incidence/	295340
36		Prevalence/	334396

No	Category	Search term	hits
37		Mortality/	48996
38		Morbidity/	33367
39		(epidemiolog* or incidence* or prevalen* or mortalit* or morbid*).tw.	2872661
40		or/34-39	3037628
41		Clinical Study/	5254
42		Case-Control Studies/	323414
43		Longitudinal Studies/	160321
44		Retrospective Studies/	1056918
45		Prospective Studies/	638016
46		Cohort Studies/	318636
47		(cohort adj (study or studies)).mp.	499659
48		(case control adj (study or studies)).mp.	361917
49		(follow up adj (study or studies)).mp.	711271
50		(observational adj (study or studies)).mp.	231490
51		(epidemiologic* adj (study or studies)).mp.	98230
52		(cross sectional adj (study or studies)).mp.	507890
53		((real word adj (study or studies or evidence)) or RWE).mp.	574
54		or/41-53	3324914
55		3 and 40	737
56		54 and 55	293
57	***Total before exclusion terms***	24 or 33 or 56	727
58	***Exclusion terms***	exp letter/ or exp editorial/ or exp case reports/ or exp published erratum/ or comment/ or historical article/	4621981
59		(exp animals/ or exp invertebrates/ or animal experimentation/ or "model, animal"/ or exp plants/ or exp fungi/) not exp humans/	5459913
60	***Total***	58 or 59	9956010
61		57 not 60	681
62		limit 61 to (english language and yr="2010 -Current")	474

Supplementary Table 3. Burden of Disease SLR Cochrane Library search strategy

No	Category	Search term	Hits
1	***Population terms***	exp Exocrine Pancreatic Insufficiency/	147
2		((pancrea* adj5 (exocrine or enzyme?) adj3 (insufficien* or deficien* or disorder? or lack* or failure? or dysfunction* or inadequa* or adequa*)) or pert).tw.	414
3		1 or 2	467
4	***Economic burden***	cost of illness/	881
5		health care costs/	2401
6		"health care economics and organizations"/	0
7		"Cost Control"/	159
8		Sick Leave/	601
9		Healthcare Financing/	8
10		Fees, Medical/	35
11		Absenteeism/	505
12		Presenteeism/	27
13		Efficiency/	363
14		Caregivers/	2581
15		"Length of Stay"/	7535
16		Critical Care/	1864
17		Hospital Costs/	642
18		"Patient Acceptance of Health Care"/	3270
19		((economic or societ* or socioeconomic or socio economic or illness or disease or patient* or caregiver* or carer*) adj3 (burden or impact or consequence?)).tw.	20122
20		(productivity or cost* or price* or pricing or pharmacoeconomic* or pharmaco-economic* or expenditure* or expens* or financ* or visit* or (length adj5 stay)).tw.	220614
21		(resource* adj2 ("use" or usage or utili* or allocat*)).tw.	7182
22		(leave adj2 (medical or sick or disability)).tw.	1900
23		or/4-22	244566
24		3 and 23	73
25	***Humanistic burden***	"Quality of Life"/	28736
26		patient-reported outcome measures/	990
27		stress, psychological/	6366
28		Patient Preference/	862
29		Caregiver Burden/	46
30		(qol or hrqol or quality of life).tw.	136830
31		((patient* or caregiver* or carer* or parent*) adj3 (satisf* or perspective* or view* or prefer* or attitude* or outcome* or burden or stress* or well being or perce* or pressur* or anxi* or depress*)).tw.	158370

No	Category	Search term	Hits
32		or/25-31	276567
33		3 and 32	114
34	***Epidemiology***	Epidemiology/	14
35		Incidence/	10972
36		Prevalence/	5314
37		Mortality/	646
38		Morbidity/	821
39		(epidemiolog* or incidence* or prevalen* or mortalit* or morbid*).tw.	289477
40		or/34-39	294045
41		Clinical Study/	0
42		Case-Control Studies/	5727
43		Longitudinal Studies/	6762
44		Retrospective Studies/	10429
45		Prospective Studies/	100983
46		Cohort Studies/	8168
47		(cohort adj (study or studies)).mp.	25697
48		(case control adj (study or studies)).mp.	14312
49		(follow up adj (study or studies)).mp.	69577
50		(observational adj (study or studies)).mp.	20506
51		(epidemiologic* adj (study or studies)).mp.	5690
52		(cross sectional adj (study or studies)).mp.	15492
53		((real word adj (study or studies or evidence)) or RWE).mp.	116
54		or/41-53	228002
55		3 and 40	96
56		54 and 55	27
57	***Total before exclusion terms***	24 or 33 or 56	159
58	***Exclusion terms***	exp letter/ or exp editorial/ or exp case reports/ or exp published erratum/ or comment/ or historical article/	0
59		(exp animals/ or exp invertebrates/ or animal experimentation/ or "model, animal"/ or exp plants/ or exp fungi/) not exp humans/	37
60	***Total***	58 or 59	37
61		57 not 60	159
62		Limit 61 to (english language and yr="2010 -Current")	104

Supplementary Table 4. Burden of Disease SLR PsycInfo search strategy

No	Category	Search term	Hits
1	***Population terms***	Exocrine Pancreatic Insufficiency.tw.	4
2		((pancrea* adj5 (exocrine or enzyme?) adj3 (insufficien* or deficien* or disorder? or lack* or failure? or dysfunction* or inadequa* or adequa*)) or pert).tw.	81
3		1 or 2	81

Supplementary Table 5. Economic models and utilities SLR Embase search strategy

No	Category	Search term	Hits
1	***Population terms***	exp exocrine pancreas insufficiency/	1,532
2		((pancrea* adj5 (exocrine or enzyme?) adj3 (insufficien* or deficien* or disorder? or lack* or failure? or dysfunction* or inadequa* or adequa*)) or pert).tw.	5,597
3		1 or 2	6,048
4	***Economic models***	economic evaluation/	18,331
5		cost effectiveness analysis/	169,921
6		cost utility analysis/	11,278
7		cost benefit analysis/	91,154
8		cost minimization analysis/	3,801
9		economic model/	2,859
10		decision tree/	18,073
11		markov chain/	8,858
12		quality adjusted life year/	32,160
13		(economic adj2 (analy* or evaluat*)).tw.	32,050
14		(cost adj2 (effective* or utilit* or minimi* or benefit)).tw.	243,140
15		(model or markov or (decision adj2 tree)).tw.	3,134,040
16		(incremental cost effectiveness ratio or icer).tw.	16,245
17		budget impact.tw.	4,946
18		(quality adjusted life year or qaly?).tw.	26,802
19		(life Years Gained or lyg?).tw.	2,768
20		or/4-19	3,476,570
21		3 and 20	418
22	***Utilities***	exp utility value/	634
23		european quality of life 5 dimensions questionnaire/	6,150
24		(utilit* or disutilit*).tw.	344,913
25		(health state* or HSUV).tw.	13,363
26		(EQ5D or EQ 5D or EuroQoL).tw.	26,464
27		(health utility index or HUI2 or HUI3 or HUI 2 or HUI 3).tw.	1,042
28		(medical outcome study or short-form or shortform or mos sf or sf6d or sf 6d or sf 6 d or sf 6 dimension or sf six or shortform six).tw.	55,260
29		patient preference.tw.	8,131
30		(Time Trade Off 18or Standard Gamble or rating$ scale).tw.	92,384
31		((mapping or crosswalk*) and (utilit* or qol or quality of life or patient reported or pro)).tw.	9,219
32		or/22-31	519,306
33		3 and 32	118
34	***Total before any exclusion and limits***	21 or 33	520

No	Category	Search term	Hits
35		exp case study/ or exp case report/ or exp letter/ or exp editorial/ or exp preliminary communication/ or exp erratum/ or exp note/	5,557,972
36		(exp animal/ or exp invertebrate/ or nonhuman/ or animal experiment/ or animal tissue/ or animal model/ or exp plant/ or exp fungus/) not (exp human/ or human tissue/)	7,411,031
37	***Total after exclusion terms***	34 not (35 or 36)	393
38	***Total after time***	limit 37 to yr="2010 -Current"	319
39	***Total after limiting to conference from the past 2/3 years***	exp conference paper/ or conference abstract/ or (conference adj (abstract or paper or review or proceeding)).pt.	5,281,514
40		limit 39 to yr="2010 - 2019"	3,060,335
41		38 not 40	185
42	***Total***	limit 41 to english language	185

Supplementary Table 6. Economic models and utilities SLR Medline search strategy

#	Category	Search term	Hits
1	***Population terms***	exp Exocrine Pancreatic Insufficiency/	2364
2		((pancrea* adj5 (exocrine or enzyme?) adj3 (insufficien* or deficien* or disorder? or lack* or failure? or dysfunction* or inadequa* or adequa*)) or pert).tw.	3312
3		1 or 2	4537
4	***Economic models***	Cost-Benefit Analysis/	90640
5		"Costs and Cost Analysis"/	50839
6		Models, Economic/	11024
7		Decision Trees/	12015
8		Markov Chains/	15795
9		Quality-Adjusted Life Years/	15093
10		(economic adj2 (analy* or evaluat*)).tw.	23036
11		(cost adj2 (effective* or utilit* or minimi* or benefit)).tw.	180144
12		(model or markov or (decision adj2 tree)).tw.	2516406
13		(incremental cost effectiveness ratio or icer).tw.	8974
14		budget impact.tw.	1795
15		(quality adjusted life year or qaly?).tw.	15487
16		(life Years Gained or lyg?).tw.	1638
17		or/4-16	2763806
18		3 and 17	223
19	***Utilities***	(utilit* or disutilit*).tw.	250421
20		(health state* or HSUV).tw.	7749
21		(EQ5D or EQ 5D or EuroQoL).tw.	14828
22		(health utility index or HUI2 or HUI3 or HUI 2 or HUI 3).tw.	691
23		(medical outcome study or short-form or shortform or mos sf or sf6d or sf 6d or sf 6 d or sf 6 dimension or sf six or shortform six).tw.	40829
24		patient preference.tw.	4711
25		(Time Trade Off or Standard Gamble or rating$ scale).tw.	62616
26		((mapping or crosswalk*) and (utilit* or qol or quality of life or patient reported or pro)).tw.	6312
27		or/19-26	367786
28		3 and 27	62
29	***Total before exclusion terms***	18 or 28	279
30	***Exclusion terms***	exp letter/ or exp editorial/ or exp case reports/ or exp published erratum/ or comment/ or historical article/	4621981
31		(exp animals/ or exp invertebrates/ or animal experimentation/ or "model, animal"/ or exp plants/ or exp fungi/) not exp humans/	5459913

#	Category	Search term	Hits
32	***Total***	30 or 31	9956010
33		29 not 32	202
34		limit 33 to (english language and yr="2010 -Current")	152

Supplementary Table 7. Economic models and utilities SLR Cochrane library search strategy

No	Category	Search term	Hits
1	***Population terms***	exp Exocrine Pancreatic Insufficiency/	147
2		((pancrea* adj5 (exocrine or enzyme?) adj3 (insufficien* or deficien* or disorder? or lack* or failure? or dysfunction* or inadequa* or adequa*)) or pert).tw.	414
3		1 or 2	467
4	***Economic models***	Cost-Benefit Analysis/	7817
5		"Costs and Cost Analysis"/	1482
6		Models, Economic/	264
7		Decision Trees/	171
8		Markov Chains/	301
9		Quality-Adjusted Life Years/	1502
10		(economic adj2 (analy* or evaluat*)).tw.	7860
11		(cost adj2 (effective* or utilit* or minimi* or benefit)).tw.	36689
12		(model or markov or (decision adj2 tree)).tw.	108857
13		(incremental cost effectiveness ratio or icer).tw.	3158
14		budget impact.tw.	348
15		(quality adjusted life year or qaly?).tw.	5612
16		(life Years Gained or lyg?).tw.	268
17		or/4-16	142296
18		3 and 17	50
19	***Utilities***	(utilit* or disutilit*).tw.	20076
20		(health state* or HSUV).tw.	1546
21		(EQ5D or EQ 5D or EuroQoL).tw.	12473
22		(health utility index or HUI2 or HUI3 or HUI 2 or HUI 3).tw.	247
23		(medical outcome study or short-form or shortform or mos sf or sf6d or sf 6d or sf 6 d or sf 6 dimension or sf six or shortform six).tw.	15628
24		patient preference.tw.	2469
25		(Time Trade Off or Standard Gamble or rating$ scale).tw.	34663
26		((mapping or crosswalk*) and (utilit* or qol or quality of life or patient reported or pro)).tw.	837
27		or/19-26	78579
28		3 and 27	26
29	***Total before exclusion terms***	18 or 28	63
30	***Exclusion terms***	exp letter/ or exp editorial/ or exp case reports/ or exp published erratum/ or comment/ or historical article/	0

No	Category	Search term	Hits
31		(exp animals/ or exp invertebrates/ or animal experimentation/ or "model, animal"/ or exp plants/ or exp fungi/) not exp humans/	37
32	***Total***	30 or 31	37
33		29 not 32	63
34		limit 33 to (english language and yr="2010 -Current") [Limit not valid in CDSR,DARE; records were retained]	42

Supplementary Table 8. Economic models and utilities SLR Econlit search strategy

No	Category	Search terms	Hits
1	***Population terms***	Exocrine Pancreatic Insufficiency.tw.	0
2		((pancrea* adj5 (exocrine or enzyme?) adj3 (insufficien* or deficien* or disorder? or lack* or failure? or dysfunction* or inadequa* or adequa*)) or pert).tw.	47
3		1 or 2	47

Supplementary Table 9. PICOTS eligibility criteria: Studies reporting burden of disease

	Inclusion Criteria	Exclusion Criteria
**Population**	All patients with EPI attributed to:<br>cystic fibrosis<br>chronic pancreatitis<br>pancreatic cancer<br>diabetes mellitus<br>or any other medically defined pancreatic disease that might require pancreatic enzyme therapy	NA
**Interventions/ comparators**	All approved or investigational pharmacotherapies (Including PERT)<br>Best supportive care or symptom/disease management	Alternative medicines (e.g., homeopathy, Chinese medicines)<br>Dietary or lifestyle interventions (e.g., smoking cessation, limiting, or avoiding alcoholic drinks, and limiting dietary fat intake)*
**Outcomes**	Economic Burden<br>Direct costs<br>Indirect costs<br>Resource use<br>Non-medical costs<br>Clinical burden<br>Incidence<br>Prevalence<br>Mortality<br>Humanistic burden<br>Patient-reported outcomes<br>Anxiety<br>Depression<br>Measures of physical function<br>EQ-5D<br>SF-36<br>Quality of life (QoL) assessment<br>EPI-Q<br>GIQLI	NA
**Time horizon**	2010 – Current	NA
**Study design**	Studies reporting economic, clinical, and humanistic burden data<br>Systematic reviews**	Editorials<br>News articles<br>Targeted literature reviews<br>Commentaries<br>Notes
**Other**	The review will only include studies published in the English language<br>No geographical limitations will be applied	Duplicates: conference abstracts that report the same data as a subsequent full text publication will be marked as duplicates and excluded<br>Nonhuman studies

*If a study encompassed dietary or lifestyle interventions in combination with pharmacotherapy, it was included. In instances where dietary and/or lifestyle interventions are the only interventions discussed, the associated publication was excluded.

**Relevant systematic reviews were included at the abstract screening stage so that their reference lists can be hand searched. These primary references were then screened using the PICOTS criteria, and if included, were extracted for reporting in the SLR report. The systematic reviews were excluded at full-text screening, to avoid duplication in reporting.

EPI: exocrine pancreatic insufficiency; EQ-5D: EuroQol five-dimension scale questionnaire; GIQLI: the Gastrointestinal Quality of Life Index; NA: not applicable; EPI-Q: the EPI questionnaire; PERT: pancreatic enzyme replacement therapy; PICOTS: population, intervention, comparator, outcomes, time horizon, study design criteria for study inclusion or exclusion; SF-36: the 36-Item Short-Form Health Survey; SLR: systematic literature review.

Supplementary Table 10. PICOTS criteria for studies reporting economic models and utilities

	Inclusion Criteria	Exclusion Criteria
**Population**	All patients with EPI attributed to:<br>cystic fibrosis<br>chronic pancreatitis<br>pancreatic cancer<br>diabetes mellitus<br>or any other medically defined pancreatic disease that might require pancreatic enzyme therapy	NA
**Interventions/ comparators**	All approved or investigational pharmacotherapies (Including PERT)<br>Best supportive care or symptom/disease management	Alternative medicines (e.g., homeopathy, Chinese medicines)<br>Dietary or lifestyle interventions (e.g., smoking cessation, limiting, or avoiding alcoholic drinks, and limiting dietary fat intake)*
**Outcomes**	Cost utility analysis**<br>Cost-effectiveness analysis**<br>Cost-benefit analysis<br>Cost minimization analysis	NA
**Time horizon**	2010 – Current	NA
**Study design**	Studies reporting cost-effectiveness data	Editorials<br>News articles<br>Targeted literature reviews<br>Commentaries<br>Notes
**Other**	The review will only include studies published in the English language<br>No geographical limitations will be applied	Duplicates: conference abstracts that report the same data as a subsequent full-text publication will be marked as duplicates and excluded<br>Nonhuman studies

*If a study encompassed dietary or lifestyle interventions that were discussed in combination with pharmacotherapy then it was included. In instances where dietary and/or lifestyle interventions are the only interventions discussed, the associated publication was excluded.

**Only de novo cost-effectiveness models were included in this review

EPI: exocrine pancreatic insufficiency; PERT: pancreatic enzyme replacement therapy; PICOTS: population, intervention, comparator, outcomes, time horizon, study design criteria for study inclusion or exclusion; NA: not applicable; SLR: systematic literature review.

Supplementary Table 11. Characteristics of studies included in the systematic literature review

Author, Year	Study design	Data collection period	Country	Previous intervention details
Observational cohort studies				
Latenstein A.E.J. 2021	Cohort study	2006–2016	The Netherlands	Pancreatic surgery (pancreatoduodenectomy or left pancreatectomy).
Kroon V.J. 2022	Cohort study (Prospective)	2014 to 2018	Netherlands	Pancreatic surgery (pancreatoduodenectomy).
Murruste M. 2022	Cohort study (Prospective)	1997 to 2021	NR	NR
Phillips A.E. 2022	Cohort study (Prospective)	30 days after enrollment	United States	NR
Reyes M. 2022	Cohort study (Prospective)	1990 to 2018	Mexico	NR
Hallac A. 2020	Cohort study (Prospective)	2005–2015	NR	Distal pancreatectomy.
Kumbhar G. 2020	Cohort study (Prospective)	2018–Present	India	NR
De la Iglesia D. 2019	Cohort study (Prospective)	2005–2015	Spain	NR
De La Iglesia-Garcia D. 2018	Cohort study (Prospective)	2005–2015	Spain	NR
Neophytou H. 2018	Cohort study (Prospective)	2005 to 2016	France	Pancreatic resection for benign tumors.
Lim P.W. 2016	Cohort study (Prospective)	January 2002 – December 2012	United States	Pancreatic resection.
Sikkens E.C.M. 2014	Cohort study (Prospective)	March 2010 – August 2012	The Netherlands	NR
Fang K. 2022	Cohort study (Retrospective)	January 2009– September 2021	China	Organ-sparing pancreatectomy for benign or low-grade malignant pancreatic tumors.
Pranger B.K. 2021	Cohort study (Retrospective)	January 2014 – December 2016	The Netherlands	Pancreatic resection.
Townsend M.J. 2021	Cohort study (Retrospective)	2011–2019	NR	NR

Author, Year	Study design	Data collection period	Country	Previous intervention details
Bartholdy A. 2020	Cohort study (Retrospective)	January 2010–December 2017	Denmark	NR
Issa Z. 2020	Cohort study (Retrospective)	NR	NR	NR
Nikolic S. 2020	Cohort study (Retrospective)	January 2004–December 2019	Sweden	NR
Scheers I. 2020	Cohort study (Retrospective)	Before January 2019	Canada	NR
Kusakabe J. 2019	Cohort study (Retrospective)	January 2000–December 2015	United States	NR
Machicado J.D. 2018	Cohort study (Retrospective)	1977 – 2016	United States	NR
Mackay T.M. 2018	Cohort study (Retrospective)	1992 – 2016	The Netherlands	Pancreatic neuroendocrine tumor resection.
Tu J. 2017	Cohort study (Retrospective)	January – April 2016	China	NR
Campbell J.A. 2016	Cohort study (Retrospective)	2009-2013	United Kingdom	NR
Roeyen G. 2016	Cohort study (Retrospective)	2009 – 2015	Belgium	Pancreatoduodenectomy.
Sudo T. 2014	Cohort study (Retrospective)	December 1996 – April 2013	Japan	Pancreatojejunostomy for CP.
Tanaka M. 2014	Cohort study (Retrospective)	June 1999 – April 2013	Japan	Surgical-drainage procedure for CP.
Garip G. 2013	Cohort study (Retrospective)	March 2003 – September 2007	Turkey	NR
Cross-sectional studies				
Gupta A. 2022	Cross-sectional study	NR	United States	Patient-administered (largely oral) drugs used to manage seven common cancer-associated symptoms or conditions, including EPI.
Anoop S. 2021	Cross-sectional study	2018 – 2019	India	NR
Lv Y. 2021	Cross-sectional study	March–July 2017	China	NR
Aksoz Z. 2020	Cross-sectional study	NR	Turkey	NR
Dieguez-Castillo C. 2020	Cross-sectional study	February 2015–June 2016	Spain	NR

Author, Year	Study design	Data collection period	Country	Previous intervention details
Kempeneers M.A. 2020	Cross-sectional study	2011–2017	The Netherlands	NR
Rosa J. 2020	Cross-sectional study	January 1994 – December 2016	Portugal	NR
Schwarzenberg S.J. 2019	Cross-sectional study	"INSPIRE: August 22 2012–March 20 2017; NAPS2: 2000–2014"	Global	NR
Smith Z.L. 2019	Cross-sectional study	January 2006-May 2016	United States	Transmural therapy for walled-off pancreatic necrosis.
Softeland E. 2019	Cross-sectional study	NR	Norway and Denmark	NR
Cabarkapa V. 2018	Cross-sectional study	October 2016 to July 2017	Serbia	NR
Marra-Lopez Valenciano C. 2018	Cross-sectional study	June 2014 – April 2015	Spain	NR
Shivaprasad C. 2015	Cross-sectional study	2012 – 2014	India	NR
Wang S. 2013	Cross-sectional study	December 2011 – November 2012	China	NR
Enrique Dominguez-Munoz J. 2012	Cross-sectional study	NR	Spain	NR
Eidt-Koch D. 2010	Cross-sectional study	April 2006–August 2006	Germany	NR
Observational studies with no specified study design				
Lamarca A. 2021	Observational study	July 2018 – October 2020	NR	NR
Sayiner Z.A. 2021	Observational study	NR	Turkey	NR
Amodio A. 2020	Observational study	January 2010–December 2016	Italy	NR
Maatman T.K. 2020	Observational study	2005–2017	United States	NR
Gelfond D. 2018	Observational study	2011–2014	United States	NR
Min M. 2018	Observational study	January 2014–December 2016	United States	NR
Dominguez-Munoz J.E. 2014	Observational study	2011	Spain	NR
Jalal 2022	Observational study (Prospective)	NR	United Kingdom	NR

Author, Year	Study design	Data collection period	Country	Previous intervention details
Keihanian T. 2022	Observational study (Prospective)	March 2018 to October 2021	NR	NR
Shah I. 2022	Observational study (Prospective)	January 2016 to April 2021	NR	NR
Yousuf A. 2022	Observational study (Prospective)	December 2019 to June 2021	India	NR
Oh M.Y. 2021	Observational study (prospective)	2008-2018	South Korea	Total pancreatectomy.
Oyon D. 2020	Observational study (Prospective)	NR	NR	NR
Koziel D. 2017	Observational study (Prospective)	2011–2012	Poland	NR
D'Haese J.G. 2014	Observational study (Prospective)	January 2006–October 2006	Germany	Creon® (pancreatin).
Vujasinovic M. 2013	Observational study (Prospective)	NR	Slovenia	NR
Halloran C.M. 2011	Observational study (prospective)	January 2000 – December 2003	United Kingdom	Pancreatectomy for neoplasia.
Cartelle A.L. 2022	Observational study (Retrospective)	January 2016–April 2021	United States	NR
Johnston P.C. 2022	Observational study (Retrospective)	February 2019 to February 2020	Northern Ireland	NR
Navaratnam J. 2022	Observational study (Retrospective)	NR	NR	NR
Thomas L. 2022	Observational study (Retrospective)	January 2010 – June 2020	India	NR
Bolasco G. 2021	Observational study (Retrospective)	31 January 2002–September 2021	Italy	Oncological pancreatic surgery.
Stoop T.F. 2020	Observational study (Retrospective)	2008–2017	Sweden	Total pancreatectomy.
Kachare S.D. 2014	Observational study (Retrospective)	2004 – 2013	United States	Pancreatic resection.
Other study designs				
Raun A.M.T. 2021	RCT	February 2019–April 2019	Denmark	NR
Masamune A. 2018	Survey	NR	Japan	NR
Johnson C.D. 2017	Qualitative Assessment	NR	Germany, France, UK	NR

INSPIRE: international study group of pediatric pancreatitis in search for a cure; NAPS2: North American pancreatitis study2; NR: not reported; RCT: randomized controlled trial.

Supplementary Table 12. Summary of clinical burden outcomes

Author, year	Study population	Outcome category	Outcome description	Timepoint/ data collection period	Subgroup	Sample size	Statistical measure	Estimate
Cartelle A.L. 2022	Disabled patients with CP	Prevalence	Prevalence of EPI	NR	CP patients (not disabled) with EPI	60	%	82.2
Cartelle A.L. 2022	Non-disabled patients with CP	Prevalence	Prevalence of EPI	NR	CP patients (disabled) with EPI	182	%	55.5
Fang K. 2022	Patients with benign or low-grade pancreatic tumors who have undergone pancreatectomy	Prevalence	Number of patients who have EPI	NR	> Total<br>> Spleen-preserving distal pancreatectomy<br>> Pylorus preserving pancreaticoduodenectomy<br>> Duodenum-preserving pancreatic head resection	> 101<br>> 25<br>> 7<br>> 7	n (%)	> 7 (6.9)<br>> 5 (20.0)<br>> 1 (14.3)<br>> 1 (14.3)
Jalal 2022	All patients referred for endoscopic ultrasound with suspected pancreatic pathology	Prevalence	Prevalence of patients with suspected sarcopenia	NR	Patients with EPI	46	%	45.1
Johnston P.C. 2022	All patients with DM or pre-diabetes	Prevalence	Prevalence of EPI in DM patients	NR	Diabetes patients with EPI	34	%	21.0
Johnston P.C. 2022	All patients with EPI	Prevalence	Prevalence of moderate EPI in DM patients	NR	Moderate EPI	9	%	5.0
Johnston P.C. 2022	All patients with EPI	Prevalence	Prevalence of severe EPI in DM patients	NR	Severe EPI	25	%	25.0

Author, year	Study population	Outcome category	Outcome description	Timepoint/ data collection period	Subgroup	Sample size	Statistical measure	Estimate
Keihanian T. 2022	Patients with new onset chronic diarrhea	Prevalence	Prevalence of EPI in patients with new onset chronic diarrhea who had an FE-1 test, N=97	NR	New chronic onset diarrhea patients with EPI	9	%	9.3
Kroon V.J. 2022	Patients prior to pancreatic surgery	Prevalence	Prevalence of EPI in patients before pancreatic surgery	NR	EPI patients	44	%	61.0
Kroon V.J. 2022	Patients prior to pancreatic surgery	Prevalence	Prevalence of EPI in patients before pancreatic surgery	NR	Moderate EPI	20	NR	NR
Kroon V.J. 2022	Patients prior to pancreatic surgery	Prevalence	Prevalence of EPI in patients before pancreatic surgery	NR	Severe EPI	8	NR	NR
Kroon V.J. 2022	All patients who survived at least two years after pancreatic surgery	Incidence	Incidence of EPI following pancreatic surgery	3 months	Patients who develop EPI following pancreatic surgery	66	%	88.0
Kroon V.J. 2022	All patients who survived at least two years after pancreatic surgery	Incidence	Incidence of EPI following pancreatic surgery	6 months	Patients who develop EPI following pancreatic surgery	59	NR	NR

Author, year	Study population	Outcome category	Outcome description	Timepoint/ data collection period	Subgroup	Sample size	Statistical measure	Estimate
Kroon V.J. 2022	All patients who survived at least two years after pancreatic surgery	Incidence	Incidence of EPI following pancreatic surgery	12 months	Patients who develop EPI following pancreatic surgery	20	N	20
Kroon V.J. 2022	All patients who survived at least two years after pancreatic surgery	Incidence	Incidence of EPI following pancreatic surgery	18 months	Patients who develop EPI following pancreatic surgery	13	n	13
Murruste M. 2022	Patients with chronic pancreatitis	Incidence	Incidence of EPI following surgery to treat chronic pancreatitis	NR	CP patients with EPI following surgery	52	%	31.3
Navaratnam J. 2022	Patients with Neuroendocrine tumor	Prevalence	Prevalence of EPI in neuroendocrine tumor patients	NR	Neuroendocrine tumor patients with EPI	153	%	59.5
Phillips A.E. 2022	Patients with acute pancreatitis	Prevalence	Prevalence of EPI following AP diagnosis	30 days following AP diagnosis	Patients with EPI	NR	%	42.3
Phillips A.E. 2022	Patients with acute pancreatitis	Prevalence	Prevalence of EPI following AP diagnosis	30 days following AP diagnosis	Patients with severe EPI (FE-1 <100ug/g)	32	%	28.8
Phillips A.E. 2022	Patients with acute pancreatitis	Prevalence	Prevalence of EPI following AP diagnosis	30 days following AP diagnosis	Patients with moderate EPI (FE-1 100-200ug/g)	15	%	13.5
Reyes M. 2022	Patients with cystic fibrosis	Prevalence	Prevalence of EPI in patients with cystic fibrosis	NR	CF patients with EPI	NR	%	91.0

Author, year	Study population	Outcome category	Outcome description	Timepoint/ data collection period	Subgroup	Sample size	Statistical measure	Estimate
Shah I. 2022	Patients with recurrent acute pancreatitis	Prevalence	Prevalence of EPI in patients with recurrent acute pancreatitis	NR	Patients with EPI and recurrent acute pancreatitis	NR	%	65.8
Shah I. 2022	Patients with chronic pancreatitis	Prevalence	Prevalence of EPI in patients without recurrent acute pancreatitis	NR	Patients with EPI and no sign of recurrent acute pancreatitis	NR	%	46.5
Thomas L. 2022	Children with CF	Prevalence	Documented EPI	January 2010 – June 2020	Children who had a pancreatic function assessment	102	n (%)	80 (78.4%)
Thomas L. 2022	Children with CF	Prevalence	EPI during follow-up	January 2010 – June 2020	Children who had a pancreatic function assessment	102	n (%)	5 (4.9%)
Thomas L. 2022	Children with CF	Prevalence	Severe EPI (FE-1 <100 µg/g)	January 2010 – June 2020	Children with PI	81	n (%)	53 (65.0%)
Thomas L. 2022	Children with CF	Prevalence	Moderate PI (FE-1 ≥100 µg/g ≤200 µg/g)	January 2010 – June 2020	Children with PI	81	n (%)	28 (35.0%)
Yousuf A. 2022	Patients with acute pancreatitis	Incidence	Incidence of EPI following an episode of AP	6 months	Patients who develop EPI following an episode of acute pancreatitis	48	n	48
Yousuf A. 2022	Patients with mild acute pancreatitis	Incidence	Incidence of EPI following an episode of AP	6 months	Patients who develop EPI following an episode of acute pancreatitis	18	n	18

Author, year	Study population	Outcome category	Outcome description	Timepoint/ data collection period	Subgroup	Sample size	Statistical measure	Estimate
Yousuf A. 2022	Patients with moderately severe acute pancreatitis	Incidence	Incidence of EPI following an episode of AP	6 months	Patients who develop EPI following an episode of acute pancreatitis	17	n	17
Yousuf A. 2022	Patients with severe acute pancreatitis	Incidence	Incidence of EPI following an episode of AP	6 months	Patients who develop EPI following an episode of acute pancreatitis	12	n	12
Anoop S. 2021	Patients with T2DM and normo-glycemic individuals	Prevalence	EPI related fat malabsorption (72 hours fecal fat > 18g)	2011-2012	Patients with T2DM, aged between 40 and 70	118	n (%)	53 (44.9)
Lamarca A. 2021	Patients with acute pancreatic cancer referred for consideration of palliative therapy	Prevalence	Number of patients who developed EPI	NR	> Diagnostic cohort<br>> Follow-up cohort	> 50<br>> 37	n (%)	> 32 (64.0)<br>> 33 (89.6)
Lv Y. 2021	Patients with T2DM	Prevalence	Number of patients with T2DM who have EPI	NR	NR	85	n (%)	16 (18.8)
Pranger B.K. 2021	PAYA patients who underwent pancreatic resection	Prevalence	Number of PAYA patients with EPI	NR	> After pancreatoduodenectomy<br>> After distal pancreatectomy	> 99<br>> 112	n (%)	> 27 (27.0)<br>> 18 (16.0)

Author, year	Study population	Outcome category	Outcome description	Timepoint/ data collection period	Subgroup	Sample size	Statistical measure	Estimate
Sayiner Z.A. 2021	Patients aged 30 years and over who had been diagnosed with type 2 DM and expressed gastrointestinal dyspeptic complaints	Prevalence	EPI	NR	DM patients	110	n (%)	0.655
Sayiner Z.A. 2021	Patients aged 30 years and over who had been diagnosed with type 2 DM and expressed gastrointestinal dyspeptic complaints	Prevalence	Mild to moderate EPI	NR	DM patients	110	n (%)	67 (60.9%)
Sayiner Z.A. 2021	Patients aged 30 years and over who had been diagnosed with type 2 DM and expressed gastrointestinal dyspeptic complaints	Prevalence	Severe EPI	NR	DM patients	110	n (%)	9 (8.2%)
Sayiner Z.A. 2021	Healthy volunteers, control group	Prevalence	Normal EPI	NR	Control group	40	n (%)	37 (92.5%)

Author, year	Study population	Outcome category	Outcome description	Timepoint/ data collection period	Subgroup	Sample size	Statistical measure	Estimate
Sayiner Z.A. 2021	Healthy volunteers, control group	Prevalence	Mild to moderate EPI	NR	Control group	40	n (%)	3 (7.5%)
Sayiner Z.A. 2021	Healthy volunteers, control group	Prevalence	Severe EPI	NR	Control group	40	n (%)	0 (0%)
Townsend M.J. 2021	Patients who experienced ICI-PI	Prevalence	Patients with ICI-PI who have EPI	2011-2019	Patients with EPI	105	n (%)	3 (3.0%)
Townsend M.J. 2021	Patients who experienced ICI-PI	Prevalence	Patients with ICI-PI manifesting as AP	2011-2019	Patients with EPI	NR	%	11.0
Townsend M.J. 2021	Patients who experienced ICI-PI	Prevalence	Patients with ICI-PI manifesting without AP	2011-2019	Patients with EPI	NR	%	0
Aksoz Z. 2020	Patients with DM	Prevalence	Prevalence of EPI	NR	NA	57	%	49.2
Aksoz Z. 2020	Healthy controls	Prevalence	Prevalence of EPI	NR	NA	36	%	16.7
Aksoz Z. 2020	Patients with DM	Prevalence	Prevalence of mild to moderate EPI	NR	NA	57	%	22.8
Aksoz Z. 2020	Healthy controls	Prevalence	Prevalence of mild to moderate EPI	NR	NA	36	%	13.8
Aksoz Z. 2020	Patients with DM	Prevalence	Prevalence of severe EPI	NR	NA	57	%	26.4
Aksoz Z. 2020	Healthy controls	Prevalence	Prevalence of severe EPI	NR	NA	36	%	2.9
Amodio A. 2020	Patients with CP	Prevalence	Patients with EPI	NR	NR	74	n (%)	36 (55.0)

Author, year	Study population	Outcome category	Outcome description	Timepoint/ data collection period	Subgroup	Sample size	Statistical measure	Estimate
Barthold y A. 2020	Patients with WON	Incidence	Patients who developed EPI	> At endoscopic intervention<br>> At discharge<br>> At follow-up	NR	125	n (%)	> 13 (10.0)<br>> 22 (18.0)<br>> 22 (18.0)
Dieguez-Castillo C. 2020	Patients with CP	Prevalence	Number of patients with CP who have EPI	NR	NR	50	n (%)	30 (60.0)
Hallac A. 2016	Patients who underwent distal pancreatectomy	Prevalence	Patients with EPI following distal pancreatectomy	NR	NR	324	n (%)	60 (18.5)
Hallac A. 2017	Patients who underwent distal pancreatectomy	Incidence	Patients with de novo EPI following distal pancreatectomy	NR	NR	302	n (%)	38 (12.6)
Hallac A. 2018	Patients who underwent distal pancreatectomy	Incidence	Incidence of preoperative EPI by final pathology	NR	> CP<br>> Introductory papillary mucinous neoplasm<br>> Mucinous cystic neoplasm<br>> Pancreatic duct adenocarcinoma<br>> Pancreatic neuroendocrine tumors	22	n (%)	> 11 (50.0)<br>> 5 (22.7)<br>> 1 (4.5)<br>> 4 (18.2)<br>> 1 (4.5)

Author, year	Study population	Outcome category	Outcome description	Timepoint/ data collection period	Subgroup	Sample size	Statistical measure	Estimate
Hallac A. 2019	Patients who underwent distal pancreatectomy	Incidence	Incidence of de novo EPI by final pathology	NR	> CP<br>> Introductory papillary mucinous neoplasm<br>> Mucinous cystic neoplasm<br>> Pancreatic duct adenocarcinoma<br>> Pancreatic neuroendocrine tumors<br>> Solid pseudopapillary tumor<br>> Pancragan glioma	38	n (%)	> 12 (31.6)<br>> 8 (21.1)<br>> 2 (5.3)<br>> 1 (2.6)<br>> 10 (26.3)<br>> 3 (7.9)<br>> 2 (5.3)
Hallac A. 2020	Patients who underwent distal pancreatectomy	Incidence	Incidence of overall EPI by final pathology	NR	> CP<br>> Introductory papillary mucinous neoplasm<br>> Mucinous cystic neoplasm<br>> Pancreatic duct adenocarcinoma<br>> Pancreatic neuroendocrine tumors<br>> Solid pseudopapillary tumor<br>> Pancragan glioma	60	n (%)	> 23 (38.3)<br>> 13 (21.7)<br>> 3 (5.0)<br>> 1 (1.7)<br>> 14 (23.3)<br>> 4 (6.7)<br>> 2 (3.3)
Issa Z. 2020	Patients with CF	Prevalence	Prevalence of EPI in patients with CF	NR	NR	113	n (%)	83
Kempeneers M.A. 2020	Patients with CP with EPI	Prevalence	Prevalence of EPI	NR	NA	987	n (%)	304 (30.8)

Author, year	Study population	Outcome category	Outcome description	Timepoint/ data collection period	Subgroup	Sample size	Statistical measure	Estimate
Kempeneers M.A. 2020	Patients with CP with potential EPI	Prevalence	Prevalence of potential EPI	NR	NA	987	n (%)	451 (45.7)
Kempeneers M.A. 2020	Patients with CP with no EPI	Prevalence	Prevalence of no EPI	NR	NA	987	n (%)	232 (22.5)
Kumbhar G. 2020	Patients with EOICP	Prevalence	Prevalence of EPI in patients not on enzyme therapy	NR	NR	37	n (%)	18 (48.65)
Maatman T.K. 2020	Patients with NP	Incidence	Number of NP patients who developed EPI	NR	NR	571	n (%)	108 (19.0)
Nikolic S. 2020	Patients with autoimmune pancreatitis	Prevalence	Prevalence of EPI in patients with autoimmune pancreatitis not receiving pharmacological treatment	Median follow-up timepoint 59.3 months	> First contact<br>> Last contact	17	n (%)	> 10 (57.1)<br>> 10 (57.1)
Nikolic S. 2020	Patients with autoimmune pancreatitis	Prevalence	Prevalence of EPI in patients with autoimmune pancreatitis receiving pharmacological treatment	Median follow-up timepoint 59.3 months	> First contact<br>> Last contact	57	n (%)	> 37 (65.1)<br>> 32 (56.8)

Author, year	Study population	Outcome category	Outcome description	Timepoint/ data collection period	Subgroup	Sample size	Statistical measure	Estimate
Oyon D. 2020	Patients with Biliary pancreatic tumors	Incidence	Incidence of EPI in patients with biliary pancreatic tumors	NR	> Overall<br>> Pancreatic ductal cell adenocarcinoma<br>> Cholangiocarcinoma<br>> Gall bladder cancer<br>> Carcinomas of the ampulla of Vater	> 165<br>> 103<br>> 44<br>> 5<br>> 13	n (%)	> 64 (38.8)<br>> 53 (51.5)<br>> 5 (11.4)<br>> 1 (20.0)<br>> 5 (38.5)
Rosa J. 2020	Patients with CF	Prevalence	Patients with EPI	NR	NR	14	n (%)	14 (100.0)
Scheers I. 2020	Patients with fibrosing pancreatitis in study population	Incidence	Patients who developed EPI	NR	NR	14	n (%)	6 (42.9)
Scheers I. 2020	Patients with fibrosing pancreatitis in literature review population	Prevalence	Patients with EPI	NR	> Fibrosing pancreatitis<br>> Autoimmune pancreatitis<br>> Autoimmune pancreatitis with Jaundice	> 45<br>> 27<br>> 18	n (%)	> 13 (30.0)<br>> 5 (19.0)<br>> 3 (17.0)
De la Iglesia D. 2019	Patients with CP	Prevalence	Prevalence of EPI in patients with CP	NR	Overall	430	n (%)	> 126 (29.3)<br>> 74 (17.2)
Kusakabe J. 2019	Patients with CP	Incidence	Incidence of patients who developed EPI post-operation	NR	NR	1717	n (%)	622 (36.2)

Author, year	Study population	Outcome category	Outcome description	Timepoint/ data collection period	Subgroup	Sample size	Statistical measure	Estimate
Schwarzenberg S.J. 2019	Pediatric patients with CP	Prevalence	Prevalence of pediatric CP patients with EPI who did not undergo total pancreatectomy	NR	NR	185	n (%)	48 (26.0)
Schwarzenberg S.J. 2019	Adult patients with CP	Prevalence	Prevalence of adult CP patients with EPI who did not undergo total pancreatectomy	NR	NR	1063	n (%)	339 (33.0)
Smith Z.L. 2019	Patients with walled-off pancreatic necrosis	Prevalence	Prevalence of EPI	1 year	Overall study population	26	%	33.0
Softeland E. 2019	Patients with DM	Prevalence	Prevalence of EPI in patients with DM	NR	NR	102	n (%)	13 (12.7)
Cabarkapa V. 2018	Patients with T1DM	Prevalence	Prevalence of EPI in patients with type 1 diabetes mellitus	NR	Mild to moderate EPI (100-200 ug/g)	NR	%	7.1
Cabarkapa V. 2018	Patients with T1DM	Prevalence	Prevalence of EPI in patients with type 1 diabetes mellitus	NR	Severe EPI (<100 ug/g)	NR	%	7.1

Author, year	Study population	Outcome category	Outcome description	Timepoint/ data collection period	Subgroup	Sample size	Statistical measure	Estimate
Cabarkapa V. 2018	Patients with T2DM	Prevalence	Prevalence of EPI in patients with type 2 diabetes mellitus	NR	Mild to moderate EPI (100-200 ug/g)	NR	%	16.1
Cabarkapa V. 2018	Patients with T2DM	Prevalence	Prevalence of EPI in patients with type 2 diabetes mellitus	NR	Severe EPI (<100 ug/g)	NR	%	4.8
Cabarkapa V. 2018	Patient without disease (control group)	Prevalence	Prevalence of EPI in patients without disease (control group)	NR	Moderate EPI	NR	%	2.5
De La Iglesia-Garcia D. 2018	Patients with CP	Mortality	Patients with EPI and CP who died during follow-up	NR	NR	126	n (%)	33 (20.6)
De La Iglesia-Garcia D. 2018	Patients with CP	Mortality	Increase in mortality rate of patients with EPI and CP	NR	NR	126	%	2.04
Gelfond D. 2018	Pediatric patients with CF	Prevalence	Pediatric patients with EPI	NR	> Who initiated PERT (overall)<br>> Who had confirmed EPI who initiated PERT<br>> Who had confirmed EPI but did not receive PERT	> 233<br>> 210	n (%)	> 210 (91.0)<br>> 205 (97.6)<br>> 6 (2.6)

Author, year	Study population	Outcome category	Outcome description	Timepoint/ data collection period	Subgroup	Sample size	Statistical measure	Estimate
Machicado J.D. 2018	Patients with Chronic Pancreatitis	Prevalence	Total patients with Exocrine insufficiency	1977 – 2006	NA	89	n (%)	27 (30.0%)
Machicado J.D. 2018	Patients with Chronic Pancreatitis	Prevalence	Patients with Exocrine insufficiency noted before or at diagnosis	1977 – 2006	NA	89	n (%)	12 (13.0%)
Machicado J.D. 2018	Patients with Chronic Pancreatitis	Prevalence	Patients with Exocrine Insufficiency noted after observation period	1977 – 2006	NA	89	n (%)	15 (17.0%)
Machicado J.D. 2018	Patients with Chronic Pancreatitis	Prevalence	Total patients with Exocrine insufficiency	1977 – 2006	Patients with Alcoholic Chronic Pancreatitis	46	n (%)	19 (41.0%)
Machicado J.D. 2018	Patients with Chronic Pancreatitis	Prevalence	Patients with Exocrine insufficiency noted before or at diagnosis	1977 – 2006	Patients with Alcoholic Chronic Pancreatitis	46	n (%)	7 (15.0%)
Machicado J.D. 2018	Patients with Chronic Pancreatitis	Prevalence	Patients with Exocrine Insufficiency noted after observation period	1977 – 2006	Patients with Alcoholic Chronic Pancreatitis	46	n (%)	12 (26.0%)
Machicado J.D. 2018	Patients with Chronic Pancreatitis	Prevalence	Total patients with Exocrine insufficiency	1977 – 2006	Patients with Non-Alcoholic Chronic Pancreatitis	43	n (%)	8 (19.0%)

Author, year	Study population	Outcome category	Outcome description	Timepoint/ data collection period	Subgroup	Sample size	Statistical measure	Estimate
Machicado J.D. 2018	Patients with Chronic Pancreatitis	Prevalence	Patients with Exocrine insufficiency noted before or at diagnosis	1977 – 2006	Patients with Non-Alcoholic Chronic Pancreatitis	43	n (%)	5 (12.0%)
Machicado J.D. 2018	Patients with Chronic Pancreatitis	Prevalence	Patients with Exocrine Insufficiency noted after observation period	1977 – 2006	Patients with Non-Alcoholic Chronic Pancreatitis	43	n (%)	3 (7.0%)
Mackay T.M. 2018	Patients with pNET who underwent pancreatic resection or enucleation	Prevalence	Total patients with EPI	1992 – 2016	NAFLD/NASH	16	n (%)	7 (43.8%)
Mackay T.M. 2018	Patients with pNET who underwent pancreatic resection or enucleation	prevalence	Total patients with EPI	1992 – 2016	no NAFLD/ NASH	65	n (%)	29(45.3%)
Mackay T.M. 2018	Patients with pNET who underwent pancreatic resection or enucleation	Prevalence	Postoperative Pancreatic Enzyme use	1992 – 2016	EPI	51	n (%)	49 (96.1%)

Author, year	Study population	Outcome category	Outcome description	Timepoint/ data collection period	Subgroup	Sample size	Statistical measure	Estimate
Mackay T.M. 2018	Patients with pNET who underwent pancreatic resection or enucleation	Prevalence	Postoperative Pancreatic Enzyme use	1992 – 2016	Non-EPI	53	n (%)	0 (0.0%)
Mackay T.M. 2018	Patients with pNET who underwent pancreatic resection or enucleation	Prevalence	EPI in patients with NF-pNET	1992 – 2016	EPI	51	n (%)	42 (82.4%)
Mackay T.M. 2018	Patients with pNET who underwent pancreatic resection or enucleation	prevalence	EPI in patients with NF-pNET	1992 – 2016	Non-EPI	53	n (%)	33 (62.3%)
Mackay T.M. 2018	Patients with pNET who underwent pancreatic resection or enucleation	Prevalence	EPI in patients with Insulinoma	1992 – 2016	EPI	51	n (%)	3 (5.9%)

Author, year	Study population	Outcome category	Outcome description	Timepoint/ data collection period	Subgroup	Sample size	Statistical measure	Estimate
Mackay T.M. 2018	Patients with pNET who underwent pancreatic resection or enucleation	Prevalence	EPI in patients with Insulinoma	1992 – 2016	Non-EPI	53	n (%)	18 (34.0%)
Mackay T.M. 2018	Patients with pNET who underwent pancreatic resection or enucleation	Prevalence	EPI in patients with Gastrinoma	1992 – 2016	EPI	51	n (%)	3 (5.9%)
Mackay T.M. 2018	Patients with pNET who underwent pancreatic resection or enucleation	Prevalence	EPI in patients with Gastrinoma	1992 – 2016	Non-EPI	53	n (%)	2 (3.8%)
Mackay T.M. 2018	Patients with pNET who underwent pancreatic resection or enucleation	Prevalence	EPI in patients with Glucagonoma	1992 – 2016	EPI	51	n (%)	1 (2.0%)

Author, year	Study population	Outcome category	Outcome description	Timepoint/ data collection period	Subgroup	Sample size	Statistical measure	Estimate
Mackay T.M. 2018	Patients with pNET who underwent pancreatic resection or enucleation	Prevalence	EPI in patients with Glucagonoma	1992 – 2016	Non-EPI	53	n (%)	0 (0.0%)
Mackay T.M. 2018	Patients with pNET who underwent pancreatic resection or enucleation	Prevalence	EPI in patients with VIPoma	1992 – 2016	EPI	51	n (%)	2 (3.9%)
Mackay T.M. 2018	Patients with pNET who underwent pancreatic resection or enucleation	Prevalence	EPI in patients with VIPoma	1992 – 2016	Non-EPI	53	n (%)	0 (0.0%)
Marra-Lopez Valenciano C. 2018	Patients with CP who had not visited a gastroenterologist or surgeon in at least 2 years	Prevalence	EPI (FE-1 levels <200µg/g)	June 2014-April 2015	NR	64	n (%)	41 (64.1%)

Author, year	Study population	Outcome category	Outcome description	Timepoint/ data collection period	Subgroup	Sample size	Statistical measure	Estimate
Marra-Lopez Valenciano C. 2018	Patients with CP who had not visited a gastroenterologist or surgeon in at least 2 years	Prevalence	Severe EPI (FE-1 levels <100µg/g)	June 2014-April 2015	NR	64	n (%)	29 (45.3%)
Masamune A. 2018	All patients with hereditary pancreatitis	Prevalence	Prevalence of EPI in patients with hereditary pancreatitis	NR	Hereditary pancreatitis patients with EPI	54	%	38.8
Min M. 2018	Patients with CP	Prevalence	> Patients who reported symptoms of EPI<br>> Patients diagnosed with EPI	NR	> Reported symptoms of EPI<br>> Positive diagnosis with EPI	> 91<br>> 77	n (%)	> 77 (84.6)<br>> 46 (60.0)
Neophytou H. 2018	All patients undergoing pancreatic resection (surgery)	Incidence	Incidence of EPI following pancreatic resection	NR	Postoperative pancreatic exocrine insufficiency	54	%	61.0
Neophytou H. 2018	Patients undergoing pancreaticoduodenectomy	Incidence	Incidence of EPI following pancreatic resection	NR	Postoperative pancreatic exocrine insufficiency	39	%	39.0
Neophytou H. 2018	Patients undergoing left pancreatectomy	Incidence	Incidence of EPI following pancreatic resection	NR	Postoperative pancreatic exocrine insufficiency	14	%	14.0

Author, year	Study population	Outcome category	Outcome description	Timepoint/ data collection period	Subgroup	Sample size	Statistical measure	Estimate
Neophytou H. 2018	Patients undergoing enucleation for benign pancreatic neoplasm	Incidence	Incidence of EPI following pancreatic resection	NR	Postoperative pancreatic exocrine insufficiency	1	%	2.0
Koziel D. 2017	Patients with severe acute pancreatitis	Prevalence	Prevalence of EPI (FE-1=<200µg/g)	NR	NA	NR	n (%)	17 (17.2)
Koziel D. 2017	Patients with mild acute pancreatitis	Prevalence	Prevalence of EPI (FE-1=<200µg/g)	NR	NA	NR	n (%)	4 (7.8)
Koziel D. 2017	Patients with recurrent severe acute pancreatitis	Prevalence	Prevalence of EPI (FE-1=<200µg/g)	NR	NA	NR	n (%)	5 (15.2)
Koziel D. 2017	Patients with recurrent mild acute pancreatitis	Prevalence	Prevalence of EPI (FE-1=<200µg/g)	NR	NA	NR	n (%)	0 (0.0)
Koziel D. 2017	Patients with severe acute pancreatitis	Prevalence	Prevalence of light EPI (FE-1=150-200µg/g)	NR	NA	NR	n (%)	5 (5.1)
Koziel D. 2017	Patients with mild acute pancreatitis	Prevalence	Prevalence of light EPI (FE-1=150-200µg/g)	NR	NA	NR	n (%)	NR
Koziel D. 2017	Patients with recurrent severe acute pancreatitis	Prevalence	Prevalence of light EPI (FE-1=150-200µg/g)	NR	NA	NR	n (%)	NR

Author, year	Study population	Outcome category	Outcome description	Timepoint/ data collection period	Subgroup	Sample size	Statistical measure	Estimate
Koziel D. 2017	Patients with recurrent mild acute pancreatitis	Prevalence	Prevalence of light EPI (FE-1=150-200µg/g)	NR	NA	NR	n (%)	NR
Koziel D. 2017	Patients with severe acute pancreatitis	Prevalence	Prevalence of moderate EPI (FE-1=100-150µg/g)	NR	NA	NR	n (%)	3 (3.0)
Koziel D. 2017	Patients with mild acute pancreatitis	Prevalence	Prevalence of moderate EPI (FE-1=100-150µg/g)	NR	NA	NR	n (%)	1 (2.0)
Koziel D. 2017	Patients with recurrent severe acute pancreatitis	Prevalence	Prevalence of moderate EPI (FE-1=100-150µg/g)	NR	NA	NR	n (%)	1 (3.0)
Koziel D. 2017	Patients with recurrent mild acute pancreatitis	Prevalence	Prevalence of moderate EPI (FE-1=100-150µg/g)	NR	NA	NR	n (%)	0 (0)
Koziel D. 2017	Patients with severe acute pancreatitis	Prevalence	Prevalence of severe EPI (FE-1=<100µg/g)	NR	NA	NR	n (%)	9 (9.1)
Koziel D. 2017	Patients with mild acute pancreatitis	Prevalence	Prevalence of severe EPI (FE-1=<100µg/g)	NR	NA	NR	n (%)	3 (5.8)
Koziel D. 2017	Patients with recurrent severe acute pancreatitis	Prevalence	Prevalence of severe EPI (FE-1=<100µg/g)	NR	NA	NR	n (%)	4 (12.1)

Author, year	Study population	Outcome category	Outcome description	Timepoint/ data collection period	Subgroup	Sample size	Statistical measure	Estimate
Koziel D. 2017	Patients with recurrent mild acute pancreatitis	Prevalence	Prevalence of severe EPI (FE-1=<100µg/g)	NR	NA	NR	n (%)	0 (0)
Tu J. 2017	Discharged AP patients invited for follow-up	Prevalence	Patients with AP who developed mild to moderate EPI	January – April 2016	Mild to moderate EPI	113	n (%)	33 (29.2)
Tu J. 2017	Discharged AP patients invited for follow-up	Prevalence	Patient with AP who developed severe EPI	January – April 2016	Patient with severe EPI	113	n (%)	7 (6.2)
Campbell J.A. 2016	Patients who had been tested for EPI with FEL-1	Prevalence	Prevalence of low fecal elastase-1 by symptom when FEL-1 >200µg/g	2009-2013	Diarrhea	1229	n	1069
Campbell J.A. 2016	Patients who had been tested for EPI with FEL-1	Prevalence	Prevalence of low fecal elastase-1 by symptom when FEL-1 >200µg/g	2009-2013	Abdominal Pain	356	n	318
Campbell J.A. 2016	Patients who had been tested for EPI with FEL-1	Prevalence	Prevalence of low fecal elastase-1 by symptom when FEL-1 >200µg/g	2009-2013	Weight loss	110	n	85

Author, year	Study population	Outcome category	Outcome description	Timepoint/ data collection period	Subgroup	Sample size	Statistical measure	Estimate
Campbell J.A. 2016	Patients who had been tested for EPI with FEL-1	Prevalence	Prevalence of low fecal elastase-1 by symptom when FEL-1 >200µg/g	2009-2013	Bloating	62	n	56
Campbell J.A. 2016	Patients who had been tested for EPI with FEL-1	Prevalence	Prevalence of low fecal elastase-1 by symptom when FEL-1 >200µg/g	2009-2013	Other	48	n	54
Campbell J.A. 2016	Patients who had been tested for EPI with FEL-1	Prevalence	Prevalence of low fecal elastase-1 by symptom when FEL-1 >200µg/g	2009-2013	Nausea	9	n	9
Campbell J.A. 2016	Patients who had been tested for EPI with FEL-1	Prevalence	Prevalence of low fecal elastase-1 by symptom when FEL-1 >200µg/g	2009-2013	Steatorrhea	7	n	2
Campbell J.A. 2016	Patients who had been tested for EPI with FEL-1	Prevalence	Prevalence of low fecal elastase-1 by symptom when FEL-1 100-200µg/g	2009-2013	Diarrhea	1229	n	68

Author, year	Study population	Outcome category	Outcome description	Timepoint/ data collection period	Subgroup	Sample size	Statistical measure	Estimate
Campbell J.A. 2016	Patients who had been tested for EPI with FEL-1	Prevalence	Prevalence of low fecal elastase-1 by symptom when FEL-1 100-200µg/g	2009-2013	Abdominal Pain	356	n	14
Campbell J.A. 2016	Patients who had been tested for EPI with FEL-1	Prevalence	Prevalence of low fecal elastase-1 by symptom when FEL-1 100-200µg/g	2009-2013	Weight loss	110	n	12
Campbell J.A. 2016	Patients who had been tested for EPI with FEL-1	Prevalence	Prevalence of low fecal elastase-1 by symptom when FEL-1 100-200µg/g	2009-2013	Bloating	62	n	3
Campbell J.A. 2016	Patients who had been tested for EPI with FEL-1	Prevalence	Prevalence of low fecal elastase-1 by symptom when FEL-1 100-200µg/g	2009-2013	Other	48	n	1
Campbell J.A. 2016	Patients who had been tested for EPI with FEL-1	Prevalence	Prevalence of low fecal elastase-1 by symptom when FEL-1 100-200µg/g	2009-2013	Nausea	9	n	0

Author, year	Study population	Outcome category	Outcome description	Timepoint/ data collection period	Subgroup	Sample size	Statistical measure	Estimate
Campbell J.A. 2016	Patients who had been tested for EPI with FEL-1	Prevalence	Prevalence of low fecal elastase-1 by symptom when FEL-1 100-200μg/g	2009-2013	Steatorrhea	7	n	1
Campbell J.A. 2016	Patients who had been tested for EPI with FEL-1	Prevalence	Prevalence of low fecal elastase-1 by symptom when FEL-1 <100μg/g	2009-2013	Diarrhea	1229	n	92
Campbell J.A. 2016	Patients who had been tested for EPI with FEL-1	Prevalence	Prevalence of low fecal elastase-1 by symptom when FEL-1 <100μg/g	2009-2013	Abdominal Pain	356	n	24
Campbell J.A. 2016	Patients who had been tested for EPI with FEL-1	Prevalence	Prevalence of low fecal elastase-1 by symptom when FEL-1 <100μg/g	2009-2013	Weight loss	110	n	13
Campbell J.A. 2016	Patients who had been tested for EPI with FEL-1	Prevalence	Prevalence of low fecal elastase-1 by symptom when FEL-1 <100μg/g	2009-2013	Bloating	62	n	3
Campbell J.A. 2016	Patients who had been tested for EPI with FEL-1	Prevalence	Prevalence of low fecal elastase-1 by symptom when FEL-1 <100μg/g	2009-2013	Other	48	n	2

Author, year	Study population	Outcome category	Outcome description	Timepoint/ data collection period	Subgroup	Sample size	Statistical measure	Estimate
Campbell J.A. 2016	Patients who had been tested for EPI with FEL-1	Prevalence	Prevalence of low fecal elastase-1 by symptom when FEL-1 <100µg/g	2009-2013	Nausea	9	n	0
Campbell J.A. 2016	Patients who had been tested for EPI with FEL-1	Prevalence	Prevalence of low fecal elastase-1 by symptom when FEL-1 <100µg/g	2009-2013	Steatorrhea	7	n	4
Campbell J.A. 2016	Patients who had been tested for EPI with FEL-1	Prevalence	Prevalence of low fecal elastase-1 by co-morbidity when FEL-1 >200µg/g	2009-2013	Alcohol	104	n	66
Campbell J.A. 2016	Patients who had been tested for EPI with FEL-1	Prevalence	Prevalence of low fecal elastase-1 by co-morbidity when FEL-1 >200µg/g	2009-2013	Diabetes mellitus	124	n	82
Campbell J.A. 2016	Patients who had been tested for EPI with FEL-1	Prevalence	Prevalence of low fecal elastase-1 by co-morbidity when FEL-1 >200µg/g	2009-2013	Irritable bowel syndrome	332	n	319
Campbell J.A. 2016	Patients who had been tested for EPI with FEL-1	Prevalence	Prevalence of low fecal elastase-1 by co-morbidity when FEL-1 >200µg/g	2009-2013	Coeliac disease	183	n	161

Author, year	Study population	Outcome category	Outcome description	Timepoint/ data collection period	Subgroup	Sample size	Statistical measure	Estimate
Campbell J.A. 2016	Patients who had been tested for EPI with FEL-1	Prevalence	Prevalence of low fecal elastase-1 by co-morbidity when FEL-1 >200µg/g	2009-2013	Inflammatory bowel disease	127	n	96
Campbell J.A. 2016	Patients who had been tested for EPI with FEL-1	Prevalence	Prevalence of low fecal elastase-1 by co-morbidity when FEL-1 >200µg/g	2009-2013	Acute pancreatitis	42	n	28
Campbell J.A. 2016	Patients who had been tested for EPI with FEL-1	Prevalence	Prevalence of low fecal elastase-1 by co-morbidity when FEL-1 >200µg/g	2009-2013	HIV	20	n	9
Campbell J.A. 2016	Patients who had been tested for EPI with FEL-1	Prevalence	Prevalence of low fecal elastase-1 by co-morbidity when FEL-1 100-200µg/g	2009-2013	Alcohol	104	n	9
Campbell J.A. 2016	Patients who had been tested for EPI with FEL-1	Prevalence	Prevalence of low fecal elastase-1 by co-morbidity when FEL-1 100-200µg/g	2009-2013	Diabetes mellitus	124	n	24

Author, year	Study population	Outcome category	Outcome description	Timepoint/ data collection period	Subgroup	Sample size	Statistical measure	Estimate
Campbell J.A. 2016	Patients who had been tested for EPI with FEL-1	Prevalence	Prevalence of low fecal elastase-1 by co-morbidity when FEL-1 100-200µg/g	2009-2013	Irritable bowel syndrome	332	n	8
Campbell J.A. 2016	Patients who had been tested for EPI with FEL-1	Prevalence	Prevalence of low fecal elastase-1 by co-morbidity when FEL-1 100-200µg/g	2009-2013	Coeliac disease	183	n	9
Campbell J.A. 2016	Patients who had been tested for EPI with FEL-1	Prevalence	Prevalence of low fecal elastase-1 by co-morbidity when FEL-1 100-200µg/g	2009-2013	Inflammatory bowel disease	127	n	14
Campbell J.A. 2016	Patients who had been tested for EPI with FEL-1	Prevalence	Prevalence of low fecal elastase-1 by co-morbidity when FEL-1 100-200µg/g	2009-2013	Acute pancreatitis	42	n	3
Campbell J.A. 2016	Patients who had been tested for EPI with FEL-1	Prevalence	Prevalence of low fecal elastase-1 by co-morbidity when FEL-1 100-200µg/g	2009-2013	HIV	20	n	7

Author, year	Study population	Outcome category	Outcome description	Timepoint/ data collection period	Subgroup	Sample size	Statistical measure	Estimate
Campbell J.A. 2016	Patients who had been tested for EPI with FEL-1	Prevalence	Prevalence of low fecal elastase-1 by symptom when FEL-1 <100μg/g	2009-2013	Alcohol	104	n	29
Campbell J.A. 2016	Patients who had been tested for EPI with FEL-1	Prevalence	Prevalence of low fecal elastase-1 by symptom when FEL-1 <100μg/g	2009-2013	Diabetes mellitus	124	n	18
Campbell J.A. 2016	Patients who had been tested for EPI with FEL-1	Prevalence	Prevalence of low fecal elastase-1 by symptom when FEL-1 <100μg/g	2009-2013	Irritable bowel syndrome	332	n	5
Campbell J.A. 2016	Patients who had been tested for EPI with FEL-1	Prevalence	Prevalence of low fecal elastase-1 by symptom when FEL-1 <100μg/g	2009-2013	Coeliac disease	183	n	13
Campbell J.A. 2016	Patients who had been tested for EPI with FEL-1	Prevalence	Prevalence of low fecal elastase-1 by symptom when FEL-1 <100μg/g	2009-2013	Inflammatory bowel disease	127	n	17
Campbell J.A. 2016	Patients who had been tested for EPI with FEL-1	Prevalence	Prevalence of low fecal elastase-1 by symptom when FEL-1 <100μg/g	2009-2013	Acute pancreatitis	42	n	11

Author, year	Study population	Outcome category	Outcome description	Timepoint/ data collection period	Subgroup	Sample size	Statistical measure	Estimate
Campbell J.A. 2016	Patients who had been tested for EPI with FEL-1	Prevalence	Prevalence of low fecal elastase-1 by symptom when FEL-1 <100µg/g	2009-2013	HIV	20	n	4
Lim P.W. 2016	Subjects who underwent pancreatic resection	Prevalence	Subjects with pre-existing exocrine insufficiency	January 2002 – December 2012	Subjects who underwent pancreatic resection	227	n	10
Lim P.W. 2016	Subjects who underwent pancreatic resection	Prevalence	Subjects who developed exocrine insufficiency over a median of 75 days (range: 0-881)	January 2002 – December 2012	Subjects who underwent pancreatic resection	217	n (%)	94 (43.0)
Lim P.W. 2016	Subjects who underwent pancreatic resection	Prevalence	Subjects who developed exocrine insufficiency within 30 days of resection	January 2002 – December 2012	Subjects who underwent pancreatic resection	94	n (%)	20 (21.0)
Lim P.W. 2016	Subjects who underwent pancreatic resection	Prevalence	Subjects who developed exocrine insufficiency between 30 and 90 days of resection	January 2002 – December 2012	Subjects who underwent pancreatic resection	94	n (%)	29 (31.0)

Author, year	Study population	Outcome category	Outcome description	Timepoint/ data collection period	Subgroup	Sample size	Statistical measure	Estimate
Lim P.W. 2016	Subjects who underwent pancreatic resection	Prevalence	Subjects who developed exocrine insufficiency after 90 days of resection	January 2002 – December 2012	Subjects who underwent pancreatic resection	94	n (%)	45 (48.0)
Lim P.W. 2016	Subjects who underwent pancreatic resection	Prevalence	Subjects who developed exocrine insufficiency after >1,000 days of resection	January 2002 – December 2012	Subjects who underwent pancreatic resection	97	n	3
Lim P.W. 2016	Subjects who underwent pancreatic resection	Prevalence	Subjects who did not develop a new exocrine insufficiency after resection	January 2002 – December 2012	Subjects who underwent pancreatic resection	227	n	120
Roeyen G. 2017	Patients undergoing PD for malignancy with reconstruction by PG	Prevalence	EPI (intake of PERT within 1 year postoperatively and/ or abnormal function test)	2009-2015	Reconstruction with PG	68	n (%)	51 (75.0)
Roeyen G. 2017	Patients undergoing PD for malignancy with reconstruction by PJ	Prevalence	EPI (intake of PERT within 1 year postoperatively and/ or abnormal function test)	2009-2015	Reconstruction with PJ	118	n (%)	54 (45.7)

Author, year	Study population	Outcome category	Outcome description	Timepoint/ data collection period	Subgroup	Sample size	Statistical measure	Estimate
Roeyen G. 2017	Patients undergoing PD for malignancy with reconstruction by PG	Prevalence	PERT within 1 year	2009-2015	Reconstruction with PG	68	n (%)	50 (75.8)
Roeyen G. 2017	Patients undergoing PD for malignancy with reconstruction by PJ	Prevalence	PERT within 1 year	2009-2015	Reconstruction with PJ	118	n (%)	45 (38.5)
Roeyen G. 2017	Patients undergoing PD for malignancy with reconstruction by PG	Prevalence	EPI in hard parenchymal remnant	2009-2015	Reconstruction with PG	68	n (%)	33 (89.1)
Roeyen G. 2017	Patients undergoing PD for malignancy with reconstruction by PJ	Prevalence	EPI in hard parenchymal remnant	2009-2015	Reconstruction with PJ	118	n (%)	22 (47.8)
Roeyen G. 2017	Patients undergoing PD for malignancy with reconstruction by PG	Prevalence	EPI in soft parenchymal remnant	2009-2015	Reconstruction with PG	68	n (%)	14 (53.8)
Roeyen G. 2017	Patients undergoing PD for malignancy with reconstruction by PJ	Prevalence	EPI in soft parenchymal remnant	2009-2015	Reconstruction with PJ	118	n (%)	14 (40.0)

Author, year	Study population	Outcome category	Outcome description	Timepoint/ data collection period	Subgroup	Sample size	Statistical measure	Estimate
Shivaprasad C. 2015	T1DM patients	Prevalence	Prevalence of pancreatic exocrine insufficiency	2012 – 2014	T1DM and FE-1 <200µg/gm	NR	%	31.4
Shivaprasad C. 2015	T2DM patients	Prevalence	Prevalence of pancreatic exocrine insufficiency	2012 – 2014	T2DM and FE-1 <200µg/gm	NR	%	29.4
Shivaprasad C. 2015	Control group	Prevalence	Prevalence of pancreatic exocrine insufficiency	2012 – 2014	Control and FE-1 <200µg/gm	NR	%	4.4
Shivaprasad C. 2015	T1DM patients	Prevalence	Prevalence of pancreatic exocrine insufficiency	2012 – 2014	T1DM and FE-1 >200µg/gm	NR	%	68.6
Shivaprasad C. 2015	T2DM patients	Prevalence	Prevalence of pancreatic exocrine insufficiency	2012 – 2014	T2DM and FE-1 >200µg/gm	NR	%	70.6
Shivaprasad C. 2015	Control group	Prevalence	Prevalence of pancreatic exocrine insufficiency	2012 – 2014	Control and FE-1 >200µg/gm	NR	%	95.6
D’Haese J.G. 2014	Patients with CP and EPI	Prevalence	Prevalence of patients who were already receiving pancreatin (Creon®) at the time of enrollment	Study enrollment	NA	294	n	206

Author, year	Study population	Outcome category	Outcome description	Timepoint/ data collection period	Subgroup	Sample size	Statistical measure	Estimate
D'Haese J.G. 2014	Patients with CP and EPI	Prevalence	Prevalence of patients with newly diagnosed EPI who were scheduled to start treatment with pancreatin (Creon®) (not previously treated with pancreatic enzyme preparations).	Study enrollment	NA	294	n	88
Dominguez-Munoz J.E. 2014	Patients at six pancreas units	Prevalence	Exocrine Pancreatic Insufficiency	2011	NR	NR	%	0.39
Kachare S.D. 2014	Patients with long-term follow-up who underwent pancreatic resection	Prevalence	Preoperative exocrine dysfunction	2004-2013	Patients with long-term follow-up who underwent pancreatic resection	161	n (%)	7 (4.3)
Kachare S.D. 2014	Patients with long-term follow-up who underwent pancreatic resection	Prevalence	Postoperative exocrine dysfunction	2004-2013	Patients with long-term follow-up who underwent pancreatic resection	161	n (%)	44 (27.3)

Author, year	Study population	Outcome category	Outcome description	Timepoint/ data collection period	Subgroup	Sample size	Statistical measure	Estimate
Sikkens E.C.M. 2014	Patients diagnosed with cancer of the pancreatic head region	Prevalence	Exocrine Pancreatic Insufficiency	March 2010 – August 2012	Time of Diagnosis	32	n (%)	21 (66.0)
Sikkens E.C.M. 2014	Patients diagnosed with cancer of the pancreatic head region	Prevalence	Exocrine Pancreatic Insufficiency of Pancreas	March 2010 – August 2012	Time of Diagnosis	32	n (%)	16 (67.0)
Sikkens E.C.M. 2014	Patients diagnosed with cancer of the pancreatic head region	Prevalence	Exocrine Pancreatic Insufficiency of Common Bile Duct	March 2010 – August 2012	Time of Diagnosis	32	n (%)	4 (80.0)
Sikkens E.C.M. 2014	Patients diagnosed with cancer of the pancreatic head region	Prevalence	Exocrine Pancreatic Insufficiency of Pancreas	March 2010 – August 2012	Time of Diagnosis	32	n (%)	1 (33.0)
Sikkens E.C.M. 2014	Patients diagnosed with cancer of the pancreatic head region	Prevalence	Exocrine Pancreatic Insufficiency	March 2010 – August 2012	End of Follow-up	24	n (%)	22 (92.0)
Sikkens E.C.M. 2014	Patients diagnosed with cancer of the pancreatic head region	Prevalence	Exocrine Pancreatic Insufficiency of Pancreas	March 2010 – August 2012	End of Follow-up	24	n (%)	17 (89.0)

Author, year	Study population	Outcome category	Outcome description	Timepoint/ data collection period	Subgroup	Sample size	Statistical measure	Estimate
Sikkens E.C.M. 2014	Patients diagnosed with cancer of the pancreatic head region	Prevalence	Exocrine Pancreatic Insufficiency of Common Bile Duct	March 2010 – August 2012	End of Follow-up	24	n (%)	3 (100.0)
Sikkens E.C.M. 2014	Patients diagnosed with cancer of the pancreatic head region	Prevalence	Exocrine Pancreatic Insufficiency of Pancreas	March 2010 – August 2012	End of Follow-up	24	n (%)	2 (100.0)
Sudo T. 2014	Patients with CP undergoing lateral pancreatojejunostomy	Prevalence	Pancreatic Exocrine Insufficiency	December 1996 – April 2013	Long-term follow-up following lateral pancreatojejunostomy	58	n (%)	30 (56.0)
Sudo T. 2014	Patients with CP undergoing lateral pancreatojejunostomy	Prevalence	New onset of diabetes	December 1996 – April 2013	Long-term follow-up following lateral pancreatojejunostomy	58	n (%)	11 (19.0)
Sudo T. 2014	Patients with CP undergoing lateral pancreatojejunostomy	Prevalence	New onset of insulin-dependent diabetes	December 1996 – April 2013	Long-term follow-up following lateral pancreatojejunostomy	58	n (%)	4 (7.0)
Tanaka M. 2014	Patients with Chronic pancreatitis	Prevalence	Pancreatic Exocrine Insufficiency	June 1999 – April 2013	All postoperative patients	28	n (%)	6 (19.0)
Tanaka M. 2014	Patients with Chronic pancreatitis	Prevalence	Pancreatic Exocrine Insufficiency	June 1999 – April 2013	Patients who underwent mFrey	21	n (%)	4 (19.0)
Tanaka M. 2014	Patients with Chronic pancreatitis	Prevalence	Pancreatic Exocrine Insufficiency	June 1999 – April 2013	Patients who underwent PJ	7	n (%)	2 (28.0)

Author, year	Study population	Outcome category	Outcome description	Timepoint/ data collection period	Subgroup	Sample size	Statistical measure	Estimate
Garip G. 2013	Patients with Acute Pancreatitis	Prevalence	Exocrine dysfunction	March 2003 – September 2007	All patients	109	%	0.14
Garip G. 2013	Patients with Acute Pancreatitis	Prevalence	Exocrine dysfunction	March 2003 – September 2007	SAP	39	n (%)	7 (17.9)
Garip G. 2013	Patients with Acute Pancreatitis	Prevalence	Exocrine dysfunction	March 2003 – September 2007	MAP	70	n (%)	8 (11.4)
Garip G. 2013	Patients with Acute Pancreatitis	Prevalence	Exocrine dysfunction	March 2003 – September 2007	NAP	30	n (%)	8 (26.6)
Garip G. 2013	Patients with Acute Pancreatitis	Prevalence	Exocrine dysfunction	March 2003 – September 2007	EAP	54	n (%)	5 (9.2)
Garip G. 2013	Patients with Acute Pancreatitis	Prevalence	Exocrine dysfunction	March 2003 – September 2007	Normal CT	25	n (%)	2 (8.0)
Vujasinovic M. 2013	Patients with Type1 DM (N=50), Patients with Type2 DM (N=50), Control group (N=50). DM duration >5 years	Prevalence	Prevalence of pancreatic exocrine insufficiency according to diabetes duration	NR	Diabetes duration: 5-10 years	45	n	2

Author, year	Study population	Outcome category	Outcome description	Timepoint/ data collection period	Subgroup	Sample size	Statistical measure	Estimate
Vujasinovic M. 2013	Patients with Type1 DM (N=50), Patients with Type2 DM (N=50), Control group (N=50). DM duration >5 years	Prevalence	Prevalence of pancreatic exocrine insufficiency according to diabetes duration	NR	Diabetes duration: 11-20 years	56	n	2
Vujasinovic M. 2013	Patients with Type1 DM (N=50), Patients with Type2 DM (N=50), Control group (N=50). DM duration >5 years	Prevalence	Prevalence of pancreatic exocrine insufficiency according to diabetes duration	NR	Diabetes duration: >20 years	49	n	4
Wang S. 2013	Critically ill adult patients without primary pancreatic diseases who were receiving early EN.	Prevalence	Moderate EPI (FE-1 ≥100 µg/g ≤200 µg/g)	December 2011 – November 2012	Critically ill adult patients without primary pancreatic diseases who were receiving early EN	563	n	191
Wang S. 2013	Critically ill adult patients without primary pancreatic diseases who were receiving early EN.	Prevalence	Severe EPI (FE-1 <100 µg/g)	December 2011 – November 2012	Critically ill adult patients without primary pancreatic diseases who were receiving early EN	563	n	103

Author, year	Study population	Outcome category	Outcome description	Timepoint/ data collection period	Subgroup	Sample size	Statistical measure	Estimate
Wang S. 2013	Critically ill adult patients without primary pancreatic diseases who were receiving early EN.	Prevalence	EPI	December 2011 – November 2012	Shock	114	%	28.9
Wang S. 2013	Critically ill adult patients without primary pancreatic diseases who were receiving early EN.	Prevalence	Non-EPI	December 2011 – November 2012	Shock	114	%	10.8
Wang S. 2013	Critically ill adult patients without primary pancreatic diseases who were receiving early EN.	Prevalence	EPI	December 2011 – November 2012	Anemia	64	%	8.8
Wang S. 2013	Critically ill adult patients without primary pancreatic diseases who were receiving early EN.	Prevalence	Non-EPI	December 2011 – November 2012	Anemia	64	%	14.1
Wang S. 2013	Critically ill adult patients without primary pancreatic diseases who were receiving early EN.	Prevalence	EPI	December 2011 – November 2012	Sepsis	78	%	17.7

Author, year	Study population	Outcome category	Outcome description	Timepoint/ data collection period	Subgroup	Sample size	Statistical measure	Estimate
Wang S. 2013	Critically ill adult patients without primary pancreatic diseases who were receiving early EN.	Prevalence	Non-EPI	December 2011 – November 2012	Sepsis	78	%	9.7
Wang S. 2013	Critically ill adult patients without primary pancreatic diseases who were receiving early EN.	Prevalence	EPI	December 2011 – November 2012	Diabetes	121	%	27.2
Wang S. 2013	Critically ill adult patients without primary pancreatic diseases who were receiving early EN.	Prevalence	Non-EPI	December 2011 – November 2012	Diabetes	121	%	15.2
Wang S. 2013	Critically ill adult patients without primary pancreatic diseases who were receiving early EN.	Prevalence	EPI	December 2011 – November 2012	Obesity	293	%	51.0
Wang S. 2013	Critically ill adult patients without primary pancreatic diseases who were receiving early EN.	Prevalence	Non-EPI	December 2011 – November 2012	Obesity	293	%	53.2

Author, year	Study population	Outcome category	Outcome description	Timepoint/ data collection period	Subgroup	Sample size	Statistical measure	Estimate
Wang S. 2013	Critically ill adult patients without primary pancreatic diseases who were receiving early EN.	Prevalence	EPI	December 2011 – November 2012	Cardiac arrest	83	%	21.1
Wang S. 2013	Critically ill adult patients without primary pancreatic diseases who were receiving early EN.	Prevalence	Non-EPI	December 2011 – November 2012	Cardiac arrest	83	%	7.8
Wang S. 2013	Critically ill adult patients without primary pancreatic diseases who were receiving early EN.	Prevalence	EPI	December 2011 – November 2012	Respiratory failure	173	%	28.6
Wang S. 2013	Critically ill adult patients without primary pancreatic diseases who were receiving early EN.	Prevalence	Non-EPI	December 2011 – November 2012	Respiratory failure	173	%	33.1
Wang S. 2013	Critically ill adult patients without primary pancreatic diseases who were receiving early EN.	Prevalence	EPI	December 2011 – November 2012	Hyperbilirubinemia	43	%	9.2

Author, year	Study population	Outcome category	Outcome description	Timepoint/ data collection period	Subgroup	Sample size	Statistical measure	Estimate
Wang S. 2013	Critically ill adult patients without primary pancreatic diseases who were receiving early EN.	Prevalence	Non-EPI	December 2011 – November 2012	Hyperbilirubinemia	43	%	6.3
Wang S. 2013	Critically ill adult patients without primary pancreatic diseases who were receiving early EN.	Prevalence	EPI	December 2011 – November 2012	Brain injury	185	%	35.7
Wang S. 2013	Critically ill adult patients without primary pancreatic diseases who were receiving early EN.	Prevalence	Non-EPI	December 2011 – November 2012	Brain injury	185	%	29.7
Wang S. 2013	Critically ill adult patients without primary pancreatic diseases who were receiving early EN.	Prevalence	EPI	December 2011 – November 2012	Hyperlactacidemia	136	%	33.0
Wang S. 2013	Critically ill adult patients without primary pancreatic diseases who were receiving early EN.	Prevalence	Non-EPI	December 2011 – November 2012	Hyperlactacidemia	136	%	14.5

Author, year	Study population	Outcome category	Outcome description	Timepoint/ data collection period	Subgroup	Sample size	Statistical measure	Estimate
Wang S. 2013	Critically ill adult patients without primary pancreatic diseases who were receiving early EN.	Prevalence	EPI	December 2011 – November 2012	Hypertriglyceridemia	114	%	22.8
Wang S. 2013	Critically ill adult patients without primary pancreatic diseases who were receiving early EN.	Prevalence	Non-EPI	December 2011 – November 2012	Hypertriglyceridemia	114	%	17.5
Wang S. 2013	Critically ill adult patients without primary pancreatic diseases who were receiving early EN.	Prevalence	EPI	December 2011 – November 2012	Mechanical Ventilation	281	%	57.1
Wang S. 2013	Critically ill adult patients without primary pancreatic diseases who were receiving early EN.	Prevalence	Non-EPI	December 2011 – November 2012	Mechanical Ventilation	281	%	42.0
Wang S. 2013	Critically ill adult patients without primary pancreatic diseases who were receiving early EN.	Prevalence	EPI	December 2011 – November 2012	CRRT	58	%	13.9

Author, year	Study population	Outcome category	Outcome description	Timepoint/ data collection period	Subgroup	Sample size	Statistical measure	Estimate
Wang S. 2013	Critically ill adult patients without primary pancreatic diseases who were receiving early EN.	Prevalence	Non-EPI	December 2011 – November 2012	CRRT	58	%	6.3
Enrique Dominguez-Munoz J. 2012	Patients with chronic pancreatitis	Prevalence	Prevalence of patients with EPI	NR	Patients with chronic pancreatitis	128	n (%)	48 (37.5)
Enrique Dominguez-Munoz J. 2012	Patients with chronic pancreatitis	Prevalence	EPI	NR	Hyperechoic foci without shadowing	120	%	35.8
Enrique Dominguez-Munoz J. 2012	Patients with chronic pancreatitis	Prevalence	EPI	NR	Stranding	120	%	35.0
Enrique Dominguez-Munoz J. 2012	Patients with chronic pancreatitis	Prevalence	EPI	NR	Lobularity	104	%	37.5
Enrique Dominguez-Munoz J. 2012	Patients with chronic pancreatitis	Prevalence	EPI	NR	Hyperechoic foci with shadowing	53	%	66.0
Enrique Dominguez-Munoz J. 2012	Patients with chronic pancreatitis	Prevalence	EPI	NR	Cysts	23	%	69.6
Enrique Dominguez-Munoz J. 2012	Patients with chronic pancreatitis	Prevalence	EPI	NR	Irregular MPD contour	106	%	38.7
Enrique Dominguez-Munoz J. 2012	Patients with chronic pancreatitis	Prevalence	EPI	NR	Hyperechoic MPD margin	114	%	36.0

Author, year	Study population	Outcome category	Outcome description	Timepoint/ data collection period	Subgroup	Sample size	Statistical measure	Estimate
Enrique Dominguez-Munoz J. 2012	Patients with chronic pancreatitis	Prevalence	EPI	NR	MPD dilation	70	%	58.6
Enrique Dominguez-Munoz J. 2012	Patients with chronic pancreatitis	Prevalence	EPI	NR	Dilated side branches	64	%	48.4
Enrique Dominguez-Munoz J. 2012	Patients with chronic pancreatitis	Prevalence	EPI	NR	MPD calculi	30	%	80.0
Eidt-Koch D. 2010	Patients with CF	Prevalence	Prevalence of EPI	NR	All patients	301	n (%)	248 (82.4)
Eidt-Koch D. 2010	Patients with CF	Prevalence	Prevalence of EPI	NR	Children aged 0 to 17 years	NR	n (%)	119 (82.1)
Eidt-Koch D. 2010	Patients with CF	Prevalence	Prevalence of EPI	NR	Adults aged ≥18 years	NR	n (%)	129 (82.7)

AP: acute pancreatitis; CF: cystic fibrosis; CP: chronic pancreatitis; DM: diabetes mellitus; EN: enteral nutrition; EOICP: early onset idiopathic chronic pancreatitis; EPI: exocrine pancreatic insufficiency; Fe-1: fecal elastase-1; ICI-PI: immune checkpoint inhibitor related pancreatic injury; mFrey: modified Frey's procedure; MPD: main pancreatic duct; NA: not applicable; NAFLD: non-alcoholic fatty liver disease; NASH: non-alcoholic steatohepatitis; NP: necrotizing pancreatitis; NR: not reported; PAYA: pediatric, adolescent, and young adult; PG: pancreaticogastrostomy; PI: pancreatic insufficiency; PJ: pancreaticojejunostomy; pNET: pancreatic neuroendocrine tumor; T1DM: type one diabetes mellitus; T2DM: type two diabetes mellitus; VIPoma: vasoactive intestinal peptide-oma; WON: walled-off necrosis.

Supplementary Table 13. Summary of humanistic burden studies

Author, Year	Study population	Specific measure	Outcome description	Timepoint	Subgroup	Samp size
Lamarca A. 2021	ACute pancreatic cancer referred for consideration of palliative therapy	Symptoms	EPI related symptoms present	July 2018–October 2020	> Flatus<br>> Weight loss<br>> Abdominal discomfort<br>> Steatorrhea	75
Latenstein A.E.J. 2021	History of pancreas surgery	EORTC QLQ-C30	EORTC QLQ-C30 function score (fatigue)	At least three years after pancreatoduodenec tomy	Fatigue (EPI)	153
Latenstein A.E.J. 2021	History of pancreas surgery	EORTC QLQ-C30	EORTC QLQ-C30 function score (fatigue)	At least three years after pancreatoduodenec tomy	Fatigue (Gen Pop)	NR
Latenstein A.E.J. 2021	History of pancreas surgery	EORTC QLQ-C30	EORTC QLQ-C30 function score (nausea/vomiting)	At least three years after pancreatoduodenec tomy	Nausea/vomiting (EPI)	153
Latenstein A.E.J. 2021	History of pancreas surgery	EORTC QLQ-C30	EORTC QLQ-C30 function score (nausea/vomiting)	At least three years after pancreatoduodenec tomy	Nausea/vomiting (Gen Pop)	NR
Latenstein A.E.J. 2021	History of pancreas surgery	EORTC QLQ-C30	EORTC QLQ-C30 function score (pain)	At least three years after pancreatoduodenec tomy	Pain (EPI)	153
Latenstein A.E.J. 2021	History of pancreas surgery	EORTC QLQ-C30	EORTC QLQ-C30 function score (pain)	At least three years after pancreatoduodenec tomy	Pain (Gen Pop)	NR
Latenstein A.E.J. 2021	History of pancreas surgery	EORTC QLQ-C30	EORTC QLQ-C30 function score (dyspnea)	At least three years after pancreatoduodenec tomy	Dyspnea (EPI)	153
Latenstein A.E.J. 2021	History of pancreas surgery	EORTC QLQ-C30	EORTC QLQ-C30 function score (dyspnea)	At least three years after pancreatoduodenec tomy	Dyspnea (Gen Pop)	NR
Latenstein A.E.J. 2021	History of pancreas surgery	EORTC QLQ-C30	EORTC QLQ-CF30 function score (Insomnia)	At least three years after pancreatoduodenec tomy	Insomnia (EPI)	153
Latenstein A.E.J. 2021	History of pancreas surgery	EORTC QLQ-C30	EORTC QLQ-C30 function score (Insomnia)	At least three years after pancreatoduodenec tomy	Insomnia (Gen Pop)	NR
Latenstein A.E.J. 2021	History of pancreas surgery	EORTC QLQ-C30	EORTC QLQ-C30 function score (appetite loss)	At least three years after pancreatoduodenec tomy	Appetite loss (EPI)	153
Latenstein A.E.J. 2021	History of pancreas surgery	EORTC QLQ-C30	EORTC QLQ-C30 function score (appetite loss)	At least three years after pancreatoduodenec tomy	Appetite loss (Gen Pop)	NR

**Author, Year**	**Study population**	**Specific measure**	**Outcome description**	**Timepoint**	**Subgroup**	**Samp size**
Latenstein A.E.J. 2021	History of pancreas surgery	EORTC QLQ-C30	EORTC QLQ-C30 function score (diarrhea)	At least three years after pancreatoduodenectomy	Diarrhea (EPI)	153
Latenstein A.E.J. 2021	History of pancreas surgery	EORTC QLQ-C30	EORTC QLQ-C30 function score (diarrhea)	At least three years after pancreatoduodenectomy	Diarrhea (Gen Pop)	NR
Latenstein A.E.J. 2021	History of pancreas surgery	EORTC QLQ-C30	EORTC QLQ-CF30 function score (constipation)	At least three years after pancreatoduodenectomy	Constipation (EPI)	153
Latenstein A.E.J. 2021	History of pancreas surgery	EORTC QLQ-C30	EORTC QLQ-C30 function score (constipation)	At least three years after pancreatoduodenectomy	Constipation (Gen Pop)	NR
Latenstein A.E.J. 2021	History of pancreas surgery	EORTC QLQ-C30	EORTC QLQ-C30 function score (constipation)	At least three years after pancreatoduodenectomy	Financial difficulties (EPI)	153
Latenstein A.E.J. 2021	History of pancreas surgery	EORTC QLQ-C30	EORTC QLQ-C30 function score (constipation)	At least three years after pancreatoduodenectomy	Financial difficulties (Gen Pop)	NR
Latenstein A.E.J. 2021	History of pancreas surgery	EORTC QLQ-C30	EORTC QLQ-C30	At least three years after pancreatoduodenectomy	Physical (EPI)	153
Latenstein A.E.J. 2021	History of pancreas surgery	EORTC QLQ-C30	EORTC QLQ-C30	At least three years after pancreatoduodenectomy	Physical (Gen Pop)	NR
Latenstein A.E.J. 2021	History of pancreas surgery	EORTC QLQ-C30	EORTC QLQ-C30	At least three years after pancreatoduodenectomy	Role (EPI)	153
Latenstein A.E.J. 2021	History of pancreas surgery	EORTC QLQ-C30	EORTC QLQ-C30	At least three years after pancreatoduodenectomy	Role (Gen Pop)	NR
Latenstein A.E.J. 2021	History of pancreas surgery	EORTC QLQ-C30	EORTC QLQ-C30	At least three years after pancreatoduodenectomy	Cognitive (EPI)	153
Latenstein A.E.J. 2021	History of pancreas surgery	EORTC QLQ-C30	EORTC QLQ-C30	At least three years after pancreatoduodenectomy	Cognitive (Gen Pop)	NR
Latenstein A.E.J. 2021	History of pancreas surgery	EORTC QLQ-C30	EORTC QLQ-C30	At least three years after pancreatoduodenectomy	Social (EPI)	153
Latenstein A.E.J. 2021	History of pancreas surgery	EORTC QLQ-C30	EORTC QLQ-C30	At least three years after pancreatoduodenectomy	Social (Gen Pop)	NR

Author, Year	Study population	Specific measure	Outcome description	Timepoint	Subgroup	Samp size
Latenstein A.E.J. 2021	History of pancreas surgery	EORTC QLQ-C30	EORTC QLQ-C30	At least three years after pancreatoduodenectomy	Emotional (EPI)	153
Latenstein A.E.J. 2021	History of pancreas surgery	EORTC QLQ-C30	EORTC QLQ-C30	At least three years after pancreatoduodenectomy	Emotional (Gen Pop)	NR
Latenstein A.E.J. 2021	History of pancreas surgery	EORTC QLQ-C30	EORTC QLQ-C30	At least three years after pancreatoduodenectomy	Global health status (EPI)	153
Latenstein A.E.J. 2021	History of pancreas surgery	EORTC QLQ-C30	EORTC QLQ-C30	At least three years after pancreatoduodenectomy	Global health status (Gen Pop)	NR
Latenstein A.E.J. 2021	History of pancreas surgery	Symptoms	Number of patients with abdominal rumbling symptoms	At least three years after pancreatoduodenectomy	Patients with CP and EPI who are receiving pancreatic enzymes	62
Latenstein A.E.J. 2021	History of pancreas surgery	Symptoms	Number of patients with abdominal cramps symptoms	At least three years after pancreatoduodenectomy	Patients with CP and EPI who are receiving pancreatic enzymes	62
Latenstein A.E.J. 2021	History of pancreas surgery	Symptoms	Number of patients with excessive flatulence symptoms	At least three years after pancreatoduodenectomy	Patients with CP and EPI who are receiving pancreatic enzymes	62
Latenstein A.E.J. 2021	History of pancreas surgery	Symptoms	Number of patients with fatty stools	At least three years after pancreatoduodenectomy	Patients with CP and EPI who are receiving pancreatic enzymes	62
Latenstein A.E.J. 2021	History of pancreas surgery	Symptoms	Number of patients with foul smelling stools	At least three years after pancreatoduodenectomy	Patients with CP and EPI who are receiving pancreatic enzymes	62
Latenstein A.E.J. 2021	History of pancreas surgery	Symptoms	Number of patients with unintentional weight loss	At least three years after pancreatoduodenectomy	Patients with CP and EPI who are receiving pancreatic enzymes	62
Latenstein A.E.J. 2021	History of pancreas surgery	Symptoms	Number of patients with no symptoms	At least three years after pancreatoduodenectomy	Patients with CP and EPI who are receiving pancreatic enzymes	62
Latenstein A.E.J. 2021	History of pancreas surgery	Symptoms	Number of patients with no abdominal pain	At least three years after pancreatoduodenectomy	Patients with CP and EPI who are receiving pancreatic enzymes	62

Author, Year	Study population	Specific measure	Outcome description	Timepoint	Subgroup	Samp size
Latenstein A.E.J. 2021	History of pancreas surgery	Symptoms	Number of patients with slight abdominal pain	At least three years after pancreatoduodenectomy	Patients with CP and EPI who are receiving pancreatic enzymes	62
Latenstein A.E.J. 2021	History of pancreas surgery	Symptoms	Number of patients with moderate abdominal pain	At least three years after pancreatoduodenectomy	Patients with CP and EPI who are receiving pancreatic enzymes	62
Latenstein A.E.J. 2021	History of pancreas surgery	Symptoms	Number of patients whose stool frequency is ≤1 times week	At least three years after pancreatoduodenectomy	Patients with CP and EPI who are receiving pancreatic enzymes	62
Latenstein A.E.J. 2021	History of pancreas surgery	Symptoms	Number of patients whose stool frequency is 1 to 3 times a week	At least three years after pancreatoduodenectomy	Patients with CP and EPI who are receiving pancreatic enzymes	62
Latenstein A.E.J. 2021	History of pancreas surgery	Symptoms	Number of patients whose stool frequency is 4 to 7 times a week	At least three years after pancreatoduodenectomy	Patients with CP and EPI who are receiving pancreatic enzymes	62
Latenstein A.E.J. 2021	History of pancreas surgery	Symptoms	Number of patients whose stool frequency is 2 to 3 times a day	At least three years after pancreatoduodenectomy	Patients with CP and EPI who are receiving pancreatic enzymes	62
Latenstein A.E.J. 2021	History of pancreas surgery	Symptoms	Number of patients whose stool frequency is ≥4 times per day	At least three years after pancreatoduodenectomy	Patients with CP and EPI who are receiving pancreatic enzymes	62
Latenstein A.E.J. 2021	History of pancreas surgery	Symptoms	Number of patients with abdominal cramps symptoms	At least three years after pancreatoduodenectomy	Patients with CP and EPI who are not receiving pancreatic enzymes	91
Latenstein A.E.J. 2021	History of pancreas surgery	Symptoms	Number of patients with excessive flatulence symptoms	At least three years after pancreatoduodenectomy	Patients with CP and EPI who are not receiving pancreatic enzymes	91
Latenstein A.E.J. 2021	History of pancreas surgery	Symptoms	Number of patients with fatty stools	At least three years after pancreatoduodenectomy	Patients with CP and EPI who are not receiving pancreatic enzymes	91
Latenstein A.E.J. 2021	History of pancreas surgery	Symptoms	Number of patients with foul smelling stools	At least three years after pancreatoduodenectomy	Patients with CP and EPI who are not receiving pancreatic enzymes	91

Author, Year	Study population	Specific measure	Outcome description	Timepoint	Subgroup	Samp size
Latenstein A.E.J. 2021	History of pancreas surgery	Symptoms	Number of patients with unintentional weight loss	At least three years after pancreatoduodenectomy	Patients with CP and EPI who are not receiving pancreatic enzymes	91
Latenstein A.E.J. 2021	History of pancreas surgery	Symptoms	Number of patients with no symptoms	At least three years after pancreatoduodenectomy	Patients with CP and EPI who are not receiving pancreatic enzymes	91
Latenstein A.E.J. 2021	History of pancreas surgery	Symptoms	Number of patients with no abdominal pain	At least three years after pancreatoduodenectomy	Patients with CP and EPI who are not receiving pancreatic enzymes	91
Latenstein A.E.J. 2021	History of pancreas surgery	Symptoms	Number of patients with slight abdominal pain	At least three years after pancreatoduodenectomy	Patients with CP and EPI who are not receiving pancreatic enzymes	91
Latenstein A.E.J. 2021	History of pancreas surgery	Symptoms	Number of patients with moderate abdominal pain	At least three years after pancreatoduodenectomy	Patients with CP and EPI who are not receiving pancreatic enzymes	91
Latenstein A.E.J. 2021	History of pancreas surgery	Symptoms	Number of patients whose stool frequency is ≤1 times week	At least three years after pancreatoduodenectomy	Patients with CP and EPI who are not receiving pancreatic enzymes	91
Latenstein A.E.J. 2021	History of pancreas surgery	Symptoms	Number of patients whose stool frequency is 1 to 3 times a week	At least three years after pancreatoduodenectomy	Patients with CP and EPI who are not receiving pancreatic enzymes	91
Latenstein A.E.J. 2021	History of pancreas surgery	Symptoms	Number of patients whose stool frequency is 4 to 7 times a week	At least three years after pancreatoduodenectomy	Patients with CP and EPI who are not receiving pancreatic enzymes	91
Latenstein A.E.J. 2021	History of pancreas surgery	Symptoms	Number of patients whose stool frequency is 2 to 3 times a day	At least three years after pancreatoduodenectomy	Patients with CP and EPI who are not receiving pancreatic enzymes	91
Latenstein A.E.J. 2021	History of pancreas surgery	Symptoms	Number of patients whose stool frequency is ≥4 times per day	At least three years after pancreatoduodenectomy	Patients with CP and EPI who are not receiving pancreatic enzymes	91
Latenstein A.E.J. 2021	History of pancreas surgery	Symptoms	Number of patients whose stool frequency is 2 to 3 times a day	At least three years after pancreatoduodenectomy	Patients with CP and EPI who are not receiving pancreatic enzymes	91

**Author, Year**	**Study population**	**Specific measure**	**Outcome description**	**Timepoint**	**Subgroup**	**Samp size**
Oh M.Y. 2021	Patients who underwent elective single stage TP	EORTC QLQ-C30, ver. 3.0	The European Organisation for Research and Treatment of Cancer Quality of Life Questionnaire Core 30 was used to assess QoL	> Preoperative<br>> 3 months postoperative<br>> 1 year postoperative	NA	30
Oh M.Y. 2021	Patients who underwent elective single stage TP	EORTC QLQ-C30, ver. 3.0	Fatigue	> Preoperative<br>> 3 months postoperative<br>> 1 year postoperative	NA	30
Oh M.Y. 2021	Patients who underwent elective single stage TP	EORTC QLQ-C30, ver. 3.0	Nausea and vomiting	> Preoperative<br>> 3 months postoperative<br>> 1 year postoperative	NA	30
Oh M.Y. 2021	Patients who underwent elective single stage TP	EORTC QLQ-C30, ver. 3.0	Pain	> Preoperative<br>> 3 months postoperative<br>> 1 year postoperative	NA	30
Oh M.Y. 2021	Patients who underwent elective single stage TP	EORTC QLQ-C30, ver. 3.0	Dyspnea	> Preoperative<br>> 3 months postoperative<br>> 1 year postoperative	NA	30
Oh M.Y. 2021	Patients who underwent elective single stage TP	EORTC QLQ-C30, ver. 3.0	Insomnia	> Preoperative<br>> 3 months postoperative<br>> 1 year postoperative	NA	30
Oh M.Y. 2021	Patients who underwent elective single stage TP	EORTC QLQ-C30, ver. 3.0	Loss of appetite	> Preoperative<br>> 3 months postoperative<br>> 1 year postoperative	NA	30
Oh M.Y. 2021	Patients who underwent elective single stage TP	EORTC QLQ-C30, ver. 3.0	Constipation	> Preoperative<br>> 3 months postoperative<br>> 1 year postoperative	NA	30
Oh M.Y. 2021	Patients who underwent elective single stage TP	EORTC QLQ-C30, ver. 3.0	Diarrhea	> Preoperative<br>> 3 months postoperative<br>> 1 year postoperative	NA	30
Oh M.Y. 2021	Patients who underwent elective single stage TP	EORTC QLQ-C30, ver. 3.0	Financial difficulties	> Preoperative<br>> 3 months postoperative<br>> 1 year postoperative	NA	30

Author, Year	Study population	Specific measure	Outcome description	Timepoint	Subgroup	Samp[illegible] size
Raun A.M.T. 2021	CF aged 0–17 years	Symptoms	Number of patients with abdominal pain symptoms during the preceding 2 wks.	Baseline	Patients aged 6–17 years	17
Raun A.M.T. 2021	CF aged 0–17 years	Symptoms	Number of patients with flatulence and foul-smelling stools symptoms during the preceding 2 wks.	Baseline	Patients aged 0–17 years	28
Raun A.M.T. 2021	CF aged 0–17 years	Symptoms	Number of patients with lack of appetite symptoms during the preceding 2 wks.	Baseline	Patients aged 0–17 years	28
Raun A.M.T. 2021	CF aged 0–17 years	Symptoms	Number of patients with severe pain during bowel movements during the preceding 2 wks.	Baseline	NR	4
Raun A.M.T. 2021	CF aged 0–17 years	Symptoms	Number of patients with abdominal distention symptoms during the preceding 2 wks.	Baseline	NR	28
Raun A.M.T. 2021	CF aged 0–17 years	Symptoms	Number of patients with normal shaped stools during the preceding 2 wks.	Baseline	NR	28
Raun A.M.T. 2021	CF aged 0–17 years	Symptoms	Number of patients with steatorrhea during the preceding 2 wks.	Baseline	NR	28
Raun A.M.T. 2021	CF aged 0–17 years	Symptoms	Number of patients with constipation during the preceding 2 wks.	Baseline	NR	28
Raun A.M.T. 2021	CF aged 0–17 years	Symptoms	Duration of pain	Baseline	Patients aged 6-17 years	5
Raun A.M.T. 2021	CF aged 0–17 years	Symptoms	Intensity of pain	Baseline	Patients aged 6-17 years	2 (ou[illegible] 4 pati[illegible] teste[illegible]
Kempeneers M.A. 2020	CP and EPI	Symptoms	Number of patients with unintentional weight loss	At inclusion in the registry	Patients with CP and EPI	302
Kempeneers M.A. 2020	CP and no EPI	Symptoms	Number of patients with unintentional weight loss	At inclusion in the registry	Patients with CP and no EPI	230
Kempeneers M.A. 2020	CP and EPI	Symptoms	Number of patients with nausea after meals	At inclusion in the registry	Patients with CP and EPI	242

Author, Year	Study population	Specific measure	Outcome description	Timepoint	Subgroup	Samp size
Kempeneers M.A. 2020	CP and no EPI	Symptoms	Number of patients with nausea after meals	At inclusion in the registry	Patients with CP and no EPI	204
Kempeneers M.A. 2020	CP and EPI	Symptoms	Number of patients with pain after meals	At inclusion in the registry	Patients with CP and EPI	242
Kempeneers M.A. 2020	CP and no EPI	Symptoms	Number of patients with pain after meals	At inclusion in the registry	Patients with CP and no EPI	204
Kempeneers M.A. 2020	CP and EPI	Symptoms	Number of patients with appetite loss	At inclusion in the registry	Patients with CP and EPI	242
Kempeneers M.A. 2020	CP and no EPI	Symptoms	Number of patients with appetite loss	At inclusion in the registry	Patients with CP and no EPI	204
Kempeneers M.A. 2020	CP and EPI	Symptoms	Number of patients with steatorrhea	At inclusion in the registry	Patients with CP and EPI	244
Kempeneers M.A. 2020	CP and no EPI	Symptoms	Number of patients with steatorrhea	At inclusion in the registry	Patients with CP and no EPI	232
Kempeneers M.A. 2020	CP and EPI	Symptoms	Defecation frequency per day	At inclusion in the registry	Patients with CP and EPI	304
Kempeneers M.A. 2020	CP and no EPI	Symptoms	Defecation frequency per day	At inclusion in the registry	Patients with CP and no EPI	58
Kempeneers M.A. 2020	CP and EPI	Symptoms	Number of patients with liquid defecation consistency	At inclusion in the registry	Patients with CP and EPI	225
Kempeneers M.A. 2020	CP and no EPI	Symptoms	Number of patients with liquid defecation consistency	At inclusion in the registry	Patients with CP and no EPI	195
Kempeneers M.A. 2020	CP and EPI	Symptoms	Number of patients with loose defecation consistency	At inclusion in the registry	Patients with CP and EPI	225
Kempeneers M.A. 2020	CP and no EPI	Symptoms	Number of patients with loose defecation consistency	At inclusion in the registry	Patients with CP and no EPI	195
Kempeneers M.A. 2020	CP and EPI	Symptoms	Number of patients with normal defecation consistency	At inclusion in the registry	Patients with CP and EPI	225
Kempeneers M.A. 2020	CP and no EPI	Symptoms	Number of patients with normal defecation consistency	At inclusion in the registry	Patients with CP and no EPI	195
Kempeneers M.A. 2020	CP and EPI	Symptoms	Number of patients with hard defecation consistency	At inclusion in the registry	Patients with CP and EPI	225

Author, Year	Study population	Specific measure	Outcome description	Timepoint	Subgroup	Samp size
Kempeneers M.A. 2020	CP and no EPI	Symptoms	Number of patients with hard defecation consistency	At inclusion in the registry	Patients with CP and no EPI	195
Kempeneers M.A. 2020	CP and EPI	Izbicki pain score	Izbicki pain score from 0 to 100	At inclusion in the registry	Patients with CP and EPI	304
Kempeneers M.A. 2020	CP and no EPI	Izbicki pain score	Izbicki pain score from 0 to 100	At inclusion in the registry	Patients with CP and no EPI	58
Kempeneers M.A. 2020	CP and EPI	SF-36	QoL score (physical component)	At inclusion in the registry	Patients with CP and EPI	304
Kempeneers M.A. 2020	CP and no EPI	SF-36	QoL score (physical component)	At inclusion in the registry	Patients with CP and no EPI	58
Kempeneers M.A. 2020	CP and EPI	SF-36	QoL score (mental component)	At inclusion in the registry	Patients with CP and EPI	304
Kempeneers M.A. 2020	CP and no EPI	SF-36	QoL score (mental component)	At inclusion in the registry	Patients with CP and no EPI	58
Kempeneers M.A. 2020	Patients with EPI receiving PERT	Symptoms	Number of patients with unintentional weight loss	At inclusion in the registry	Patients with EPI receiving PERT	264
Kempeneers M.A. 2020	Patients with EPI not receiving PERT	Symptoms	Number of patients with unintentional weight loss	At inclusion in the registry	Patients with EPI not receiving PERT	38
Kempeneers M.A. 2020	Patients with EPI receiving PERT	Symptoms	Number of patients with nausea after meals	At inclusion in the registry	Patients with EPI receiving PERT	205
Kempeneers M.A. 2020	Patients with EPI not receiving PERT	Symptoms	Number of patients with nausea after meals	At inclusion in the registry	Patients with EPI not receiving PERT	37
Kempeneers M.A. 2020	Patients with EPI receiving PERT	Symptoms	Number of patients with pain after meals	At inclusion in the registry	Patients with EPI receiving PERT	205
Kempeneers M.A. 2020	Patients with EPI not receiving PERT	Symptoms	Number of patients with pain after meals	At inclusion in the registry	Patients with EPI not receiving PERT	37
Kempeneers M.A. 2020	Patients with EPI receiving PERT	Symptoms	Number of patients with appetite loss	At inclusion in the registry	Patients with EPI receiving PERT	205
Kempeneers M.A. 2020	Patients with EPI not receiving PERT	Symptoms	Number of patients with appetite loss	At inclusion in the registry	Patients with EPI not receiving PERT	37
Kempeneers M.A. 2020	Patients with EPI receiving PERT	Symptoms	Number of patients with steatorrhea	At inclusion in the registry	Patients with EPI receiving PERT	206
Kempeneers M.A. 2020	Patients with EPI not receiving PERT	Symptoms	Number of patients with steatorrhea	At inclusion in the registry	Patients with EPI not receiving PERT	38
Kempeneers M.A. 2020	Patients with EPI receiving PERT	Symptoms	Defecation frequency per day	At inclusion in the registry	Patients with EPI receiving PERT	266

Author, Year	Study population	Specific measure	Outcome description	Timepoint	Subgroup	Samp[illegible] size
Kempeneers M.A. 2020	Patients with EPI not receiving PERT	Symptoms	Defecation frequency per day	At inclusion in the registry	Patients with EPI not receiving PERT	38
Kempeneers M.A. 2020	Patients with EPI receiving PERT	Symptoms	Number of patients with liquid defecation consistency	At inclusion in the registry	Patients with EPI receiving PERT	192
Kempeneers M.A. 2020	Patients with EPI not receiving PERT	Symptoms	Number of patients with liquid defecation consistency	At inclusion in the registry	Patients with EPI not receiving PERT	33
Kempeneers M.A. 2020	Patients with EPI receiving PERT	Symptoms	Number of patients with loose defecation consistency	At inclusion in the registry	Patients with EPI receiving PERT	192
Kempeneers M.A. 2020	Patients with EPI not receiving PERT	Symptoms	Number of patients with loose defecation consistency	At inclusion in the registry	Patients with EPI not receiving PERT	33
Kempeneers M.A. 2020	Patients with EPI receiving PERT	Symptoms	Number of patients with normal defecation consistency	At inclusion in the registry	Patients with EPI receiving PERT	192
Kempeneers M.A. 2020	Patients with EPI not receiving PERT	Symptoms	Number of patients with normal defecation consistency	At inclusion in the registry	Patients with EPI not receiving PERT	33
Kempeneers M.A. 2020	Patients with EPI receiving PERT	Symptoms	Number of patients with hard defecation consistency	At inclusion in the registry	Patients with EPI receiving PERT	192
Kempeneers M.A. 2020	Patients with EPI not receiving PERT	Symptoms	Number of patients with hard defecation consistency	At inclusion in the registry	Patients with EPI not receiving PERT	33
Kempeneers M.A. 2020	Patients with EPI receiving PERT	Izbicki pain score	Score can range from 0 to 100	At inclusion in the registry	Patients with EPI receiving PERT	266
Kempeneers M.A. 2020	Patients with EPI not receiving PERT	Izbicki pain score	Score can range from 0 to 101	At inclusion in the registry	Patients with EPI not receiving PERT	38
Kempeneers M.A. 2020	Patients with EPI receiving PERT	SF-36	QoL score (physical component)	At inclusion in the registry	Patients with EPI receiving PERT	266
Kempeneers M.A. 2020	Patients with EPI not receiving PERT	SF-36	QoL score (physical component)	At inclusion in the registry	Patients with EPI not receiving PERT	38
Kempeneers M.A. 2020	Patients with EPI receiving PERT	SF-36	QoL score (mental component)	At inclusion in the registry	Patients with EPI receiving PERT	266
Kempeneers M.A. 2020	Patients with EPI not receiving PERT	SF-36	QoL score (mental component)	At inclusion in the registry	Patients with EPI not receiving PERT	38

Author, Year	Study population	Specific measure	Outcome description	Timepoint	Subgroup	Samp size
Stoop T.F. 2020	Patients who underwent TP between 2008 and 2017 and were eligible for participation in the study	Questionnaire (supplementary digital content 1)	Number of patients with diarrhea symptoms in the past week	At time of answering the questionnaire	NA	53
Stoop T.F. 2020	Patients who underwent TP between 2008 and 2017 and were eligible for participation in the study	Questionnaire (supplementary digital content 1)	Duration of diarrhea symptoms in the past week	At time of answering the questionnaire	Patients who reported that they had experienced diarrhea symptoms	34
Stoop T.F. 2020	Patients who underwent TP between 2008 and 2017 and were eligible for participation in the study	Questionnaire (supplementary digital content 1)	Number of times patients had to go to the toilet per day	At time of answering the questionnaire	Patients who reported that they had experienced diarrhea symptoms	34
Stoop T.F. 2020	Patients who underwent TP between 2008 and 2017 and were eligible for participation in the study	Questionnaire (supplementary digital content 1)	Number of patients with steatorrhea in the past week	At time of answering the questionnaire	NA	53
Stoop T.F. 2020	Patients who underwent TP between 2008 and 2017 and were eligible for participation in the study	Questionnaire (supplementary digital content 1)	Number of patients with steatorrhea in the past week	At time of answering the questionnaire	NA	53
Stoop T.F. 2020	Patients who underwent TP between 2008 and 2017 and were eligible for participation in the study	Questionnaire (supplementary digital content 1)	Duration of steatorrhea in the past week	At time of answering the questionnaire	Patients who reported that they had experienced steatorrhea	27
Stoop T.F. 2020	Patients who underwent TP between 2008 and 2017 and were eligible for participation in the study	Questionnaire (supplementary digital content 1)	Number of patients whose diarrhea and steatorrhea symptoms increased in frequency	At follow-up	NA	53
Stoop T.F. 2020	Patients who underwent TP between 2008 and 2017 and were eligible for participation in the study	Questionnaire (supplementary digital content 1)	Number of patients whose diarrhea and steatorrhea symptoms remained stable in frequency	At follow-up	NA	53

Author, Year	Study population	Specific measure	Outcome description	Timepoint	Subgroup	Samp size
Stoop T.F. 2020	Patients who underwent TP between 2008 and 2017 and were eligible for participation in the study	Questionnaire (supplementary digital content 1)	Number of patients whose diarrhea and steatorrhea symptoms decreased in frequency	At follow-up	NA	53
Stoop T.F. 2020	Patients who underwent TP between 2008 and 2017 and were eligible for participation in the study	BSFS	Stool consistency as scored using the BSFS	NR	NA	NR
Smith Z.L. 2019	Patients undergoing endoscopic therapy for walled-off pancreatic necrosis with EPI	SF-36	Physical functioning SF-36 domain score	NR	Patients with EPI	14
Smith Z.L. 2019	Patients undergoing endoscopic therapy for walled-off pancreatic necrosis with EPI	SF-36	Physical role SF-36 domain score	NR	Patients with EPI	14
Smith Z.L. 2019	Patients undergoing endoscopic therapy for walled-off pancreatic necrosis with EPI	SF-36	Bodily pain SF-36 domain score	NR	Patients with EPI	14
Smith Z.L. 2019	Patients undergoing endoscopic therapy for walled-off pancreatic necrosis with EPI	SF-36	General health SF-36 domain score	NR	Patients with EPI	14
Smith Z.L. 2019	Patients undergoing endoscopic therapy for walled-off pancreatic necrosis with EPI	SF-36	Vitality SF-36 domain score	NR	Patients with EPI	14
Smith Z.L. 2019	Patients undergoing endoscopic therapy for walled-off pancreatic necrosis with EPI	SF-36	Social functioning SF-36 domain score	NR	Patients with EPI	14
Smith Z.L. 2019	Patients undergoing endoscopic therapy for walled-off pancreatic necrosis with EPI	SF-36	Emotional role SF-36 domain score	NR	Patients with EPI	14

Author, Year	Study population	Specific measure	Outcome description	Timepoint	Subgroup	Samp[illegible] size
Smith Z.L. 2019	Patients undergoing endoscopic therapy for walled-off pancreatic necrosis with EPI	SF-36	Mental health SF-36 domain score	NR	Patients with EPI	14
Marra-Lopez Valenciano C. 2018	CP Patients with EPI	QLQ-C30	Results of the symptom scale and single items of the QLQ-C30 QoL questionnaire (EPI vs non-EPI).	NR	Physical functioning (non-EPI)	22
Marra-Lopez Valenciano C. 2018	CP Patients without EPI	QLQ-C30	Results of the symptom scale and single items of the QLQ-C30 QoL questionnaire (EPI vs non-EPI).	NR	Role functioning (EPI)	41
Marra-Lopez Valenciano C. 2018	Overall population (with and without EPI)	QLQ-C30	Results of the symptom scale and single items of the QLQ-C30 QoL questionnaire (EPI vs non-EPI).	NR	Role functioning (non-EPI)	22
Marra-Lopez Valenciano C. 2018	Overall population (with and without EPI)	QLQ-C30	Results of the symptom scale and single items of the QLQ-C30 QoL questionnaire (EPI vs non-EPI).	NR	Emotional functioning (EPI)	41
Marra-Lopez Valenciano C. 2018	Overall population (with and without EPI)	QLQ-C30	Results of the symptom scale and single items of the QLQ-C30 QoL questionnaire (EPI vs non-EPI).	NR	Emotional functioning (non-EPI)	22
Marra-Lopez Valenciano C. 2018	Overall population (with and without EPI)	QLQ-C30	Results of the symptom scale and single items of the QLQ-C30 QoL questionnaire (EPI vs non-EPI).	NR	Cognitive functioning (EPI)	41
Marra-Lopez Valenciano C. 2018	Overall population (with and without EPI)	QLQ-C30	Results of the symptom scale and single items of the QLQ-C30 QoL questionnaire (EPI vs non-EPI).	NR	Cognitive functioning (non-EPI)	22
Marra-Lopez Valenciano C. 2018	Overall population (with and without EPI)	QLQ-C30	Results of the symptom scale and single items of the QLQ-C30 QoL questionnaire (EPI vs non-EPI).	NR	Social functioning (EPI)	41

Author, Year	Study population	Specific measure	Outcome description	Timepoint	Subgroup	Samp size
Marra-Lopez Valenciano C. 2018	Overall population (with and without EPI)	QLQ-C30	Results of the symptom scale and single items of the QLQ-C30 QoL questionnaire (EPI vs non-EPI).	NR	Social functioning (non-EPI)	22
Marra-Lopez Valenciano C. 2018	Overall population (with and without EPI)	QLQ-C30	Results of the symptom scale and single items of the QLQ-C30 QoL questionnaire (EPI vs non-EPI).	NR	Global health status (EPI)	41
Marra-Lopez Valenciano C. 2018	Overall population (with and without EPI)	QLQ-C30	Results of the symptom scale and single items of the QLQ-C30 QoL questionnaire (EPI vs non-EPI).	NR	Global health status (non-EPI)	22
Marra-Lopez Valenciano C. 2018	Overall population (with and without EPI)	QLQ-C30	Results of the symptom scale and single items of the QLQ-C30 QoL questionnaire (EPI vs non-EPI).	NR	Fatigue (EPI)	41
Marra-Lopez Valenciano C. 2018	Overall population (with and without EPI)	QLQ-C30	Results of the symptom scale and single items of the QLQ-C30 QoL questionnaire (EPI vs non-EPI).	NR	Fatigue (non-EPI)	22
Marra-Lopez Valenciano C. 2018	Overall population (with and without EPI)	QLQ-C30	Results of the symptom scale and single items of the QLQ-C30 QoL questionnaire (EPI vs non-EPI).	NR	Nausea/vomiting (EPI)	41
Marra-Lopez Valenciano C. 2018	Overall population (with and without EPI)	QLQ-C30	Results of the symptom scale and single items of the QLQ-C30 QoL questionnaire (EPI vs non-EPI).	NR	Nausea/vomiting (non-EPI)	22
Marra-Lopez Valenciano C. 2018	Overall population (with and without EPI)	QLQ-C30	Results of the symptom scale and single items of the QLQ-C30 QoL questionnaire (EPI vs non-EPI).	NR	Pain (EPI)	41
Marra-Lopez Valenciano C. 2018	Overall population (with and without EPI)	QLQ-C30	Results of the symptom scale and single items of the QLQ-C30 QoL questionnaire (EPI vs non-EPI).	NR	Pain (non-EPI)	22

Author, Year	Study population	Specific measure	Outcome description	Timepoint	Subgroup	Samp[illegible] size
Marra-Lopez Valenciano C. 2018	Overall population (with and without EPI)	QLQ-C30	Results of the symptom scale and single items of the QLQ-C30 QoL questionnaire (EPI vs non-EPI).	NR	Dyspnea (EPI)	41
Marra-Lopez Valenciano C. 2018	Overall population (with and without EPI)	QLQ-C30	Results of the symptom scale and single items of the QLQ-C30 QoL questionnaire (EPI vs non-EPI).	NR	Dyspnea (non-EPI)	22
Marra-Lopez Valenciano C. 2018	Overall population (with and without EPI)	QLQ-C30	Results of the symptom scale and single items of the QLQ-C30 QoL questionnaire (EPI vs non-EPI).	NR	Insomnia (EPI)	41
Marra-Lopez Valenciano C. 2018	Overall population (with and without EPI)	QLQ-C30	Results of the symptom scale and single items of the QLQ-C30 QoL questionnaire (EPI vs non-EPI).	NR	Insomnia (non-EPI)	22
Marra-Lopez Valenciano C. 2018	Overall population (with and without EPI)	QLQ-C30	Results of the symptom scale and single items of the QLQ-C30 QoL questionnaire (EPI vs non-EPI).	NR	Appetite loss (EPI)	41
Marra-Lopez Valenciano C. 2018	Overall population (with and without EPI)	QLQ-C30	Results of the symptom scale and single items of the QLQ-C30 QoL questionnaire (EPI vs non-EPI).	NR	Appetite loss (non-EPI)	22
Marra-Lopez Valenciano C. 2018	Overall population (with and without EPI)	QLQ-C30	Results of the symptom scale and single items of the QLQ-C30 QoL questionnaire (EPI vs non-EPI).	NR	Constipation (EPI)	41
Marra-Lopez Valenciano C. 2018	Overall population (with and without EPI)	QLQ-C30	Results of the symptom scale and single items of the QLQ-C30 QoL questionnaire (EPI vs non-EPI).	NR	Constipation (non-EPI)	22
Marra-Lopez Valenciano C. 2018	Overall population (with and without EPI)	QLQ-C30	Results of the symptom scale and single items of the QLQ-C30 QoL questionnaire (EPI vs non-EPI).	NR	Diarrhea (EPI)	41

Author, Year	Study population	Specific measure	Outcome description	Timepoint	Subgroup	Samp size
Marra-Lopez Valenciano C. 2018	Overall population (with and without EPI)	QLQ-C30	Results of the symptom scale and single items of the QLQ-C30 QoL questionnaire (EPI vs non-EPI).	NR	Diarrhea (non-EPI)	22
Marra-Lopez Valenciano C. 2018	Overall population (with and without EPI)	QLQ-C30	Results of the symptom scale and single items of the QLQ-C30 QoL questionnaire (EPI vs non-EPI).	NR	Financial difficulties (EPI)	41
Marra-Lopez Valenciano C. 2018	Overall population (with and without EPI)	QLQ-C30	Results of the symptom scale and single items of the QLQ-C30 QoL questionnaire (EPI vs non-EPI).	NR	Financial difficulties (non-EPI)	22
Johnson C.D. 2017	Patients with EPI and either CP or CF	Symptoms	Experienced pain	NR	NA	61
Johnson C.D. 2017	Patients with EPI and either CP or CF	Symptoms	Abdominal pain	NR	NA	61
Johnson C.D. 2017	Patients with EPI and either CP or CF	Symptoms	Bloating	NR	NA	61
Johnson C.D. 2017	Patients with EPI and either CP or CF	Symptoms	Stomach noises	NR	NA	61
Johnson C.D. 2017	Patients with EPI and either CP or CF	Symptoms	High flatulence	NR	NA	61
Johnson C.D. 2017	Patients with EPI and either CP or CF	Symptoms	Trapped wind	NR	NA	61
Johnson C.D. 2017	Patients with EPI and either CP or CF	Symptoms	Constipation	NR	NA	61
Johnson C.D. 2017	Patients with EPI and either CP or CF	Symptoms	Increased bowel movement	NR	NA	61
Johnson C.D. 2017	Patients with EPI and either CP or CF	Symptoms	Bowel urgency	NR	NA	61
Johnson C.D. 2017	Patients with EPI and either CP or CF	Symptoms	Diarrhea	NR	NA	61
Johnson C.D. 2017	Patients with EPI and either CP or CF	Symptoms	Fatty stools	NR	NA	61
Johnson C.D. 2017	Patients with EPI and either CP or CF	Symptoms	Change in stool color	NR	NA	61
Johnson C.D. 2017	Patients with EPI and either CP or CF	Symptoms	Unusual or strong odor	NR	NA	61

Author, Year	Study population	Specific measure	Outcome description	Timepoint	Subgroup	Samp size
Johnson C.D. 2017	Patients with EPI and either CP or CF	Symptoms	Nausea	NR	NA	61
Johnson C.D. 2017	Patients with EPI and either CP or CF	Symptoms	Vomiting	NR	NA	61
Johnson C.D. 2017	Patients with EPI and either CP or CF	Symptoms	Nausea and vomiting	NR	NA	61
Johnson C.D. 2017	Patients with EPI and either CP or CF	Symptoms	Weight loss	NR	NA	61
Johnson C.D. 2017	Patients with EPI and either CP or CF	Symptoms	Loss of appetite	NR	NA	61
Johnson C.D. 2017	Patients with EPI and either CP or CF	Symptoms	Tiredness	NR	NA	61
D'Haese J.G. 2014	Patients with CP and EPI	Symptoms	Malabsorption	> Baseline<br>> 6 months<br>> 12 months	Overall cohort (patients with EPI and CP)	294
D'Haese J.G. 2014	Patients with CP and EPI	Symptoms	Diarrhea/steatorrhea	> Baseline<br>> 6 months<br>> 12 months	Overall cohort (patients with EPI and CP)	294
D'Haese J.G. 2014	Patients with CP and EPI	Symptoms	Weight loss	> Baseline<br>> 6 months<br>> 12 months	Overall cohort (patients with EPI and CP)	294
D'Haese J.G. 2014	Patients with CP and EPI	Symptoms	Meteorism	> Baseline<br>> 6 months<br>> 12 months	Overall cohort (patients with EPI and CP)	294
D'Haese J.G. 2014	Patients with CP and EPI	Symptoms	Dyspepsia	> Baseline<br>> 6 months<br>> 12 months	Overall cohort (patients with EPI and CP)	294
D'Haese J.G. 2014	Patients with CP and EPI	Symptoms	Recurrent pain	> Baseline<br>> 6 months<br>> 12 months	Overall cohort (patients with EPI and CP)	294
D'Haese J.G. 2014	Patients with CP and EPI	Symptoms	Nausea	> Baseline<br>> 6 months<br>> 12 months	Overall cohort (patients with EPI and CP)	294
D'Haese J.G. 2014	Patients with CP and EPI	Symptoms	Vomiting	> Baseline<br>> 6 months<br>> 12 months	Overall cohort (patients with EPI and CP)	294
D'Haese J.G. 2014	Patients with CP and EPI	GIQLI	Total score	> Baseline<br>> 6 months<br>> 12 months	Overall cohort (patients with EPI and CP)	294
D'Haese J.G. 2014	Patients with CP and EPI	GIQLI	Symptom scale	> Baseline<br>> 6 months<br>> 12 months	Overall cohort (patients with EPI and CP)	294
D'Haese J.G. 2014	Patients with CP and EPI	GIQLI	Physical function	> Baseline<br>> 6 months<br>> 12 months	Overall cohort (patients with EPI and CP)	294
D'Haese J.G. 2014	Patients with CP and EPI	GIQLI	Social function	> Baseline<br>> 6 months<br>> 12 months	Overall cohort (patients with EPI and CP)	294
D'Haese J.G. 2014	Patients with CP and EPI	GIQLI	Emotion	> Baseline<br>> 6 months<br>> 12 months	Overall cohort (patients with EPI and CP)	294

**Author, Year**	**Study population**	**Specific measure**	**Outcome description**	**Timepoint**	**Subgroup**	**Samp[illegible] size**
D'Haese J.G. 2014	Patients with CP and EPI	GIQLI	Medical treatment	> Baseline<br>> 6 months<br>> 12 months	Overall cohort (patients with EPI and CP)	294
Halloran C.M. 2011	Patients who underwent pancreatectomy for neoplasia	EORTC QLQ-C30 (version 1)	QoL upon enrollment	At recruitment	NA	40
Halloran C.M. 2011	Patients who underwent pancreatectomy for neoplasia	EORTC QLQ-C30 (version 1)	QoL after 8 weeks of treatment	8 weeks	NA	40
Halloran C.M. 2011	Patients who underwent pancreatectomy for neoplasia	EORTC QLQ-C30 (version 1)	QoL after 3 months of treatment	3 months	NA	40
Halloran C.M. 2011	Patients who underwent pancreatectomy for neoplasia	EORTC QLQ-C30 (version 1)	QoL after 6 months of treatment	6 months	NA	40
Halloran C.M. 2011	Patients who underwent pancreatectomy for neoplasia	EORTC QLQ-C30 (version 1)	QoL after 12 months of treatment	12 months	NA	40

BSFS: Bristol stool form scale; CF: cystic fibrosis; CP: chronic pancreatitis; EORTC QLQ-C30: European Organisation for Research and Treatment of Cancer quality of life questionnaire-C30; EPI: exocrine pancreatic insufficiency; GIQLI: gastrointestinal quality of life questionnaire; IQR: interquartile range; NA: not applicable; NR: not reported; PERT: pancreatic enzyme replacement therapy; QoL: quality of life; SD: standard deviation; SF-36: 36-item short-form health survey; TP: total pancreatectomy; ver.: version.

Supplementary Table 14. Summary of economic burden outcomes

Author, year	Study population	Cost category	Cost description	Timepoint	Subgroup	Sample size
Cartelle A.L. 2022	Disabled patients with CP (82% of cohort have EPI)	Resource use	Rate of ED visits for opioid pain medication	NR	CP patients (disabled) with EPI	73
Cartelle A.L. 2022	Non-disabled patients with CP (82% of cohort have EPI)	Resource use	Rate of ED visits for opioid pain medication	NR	CP patients (not disabled) with EPI	331
Cartelle A.L. 2022	Disabled patients with CP (82% of cohort have EPI)	Resource use	Number of flares requiring hospitalization	NR	CP patients (disabled) with EPI	73
Cartelle A.L. 2022	Non-disabled patients with CP (82% of cohort have EPI)	Resource use	Number of flares requiring hospitalization	NR	CP patients (not disabled) with EPI	331
Cartelle A.L. 2022	Disabled patients with CP (82% of cohort have EPI)	Resource use	Celiac blocks	NR	CP patients (disabled) with EPI	73
Cartelle A.L. 2022	Non-disabled patients with CP (82% of cohort have EPI)	Resource use	Celiac blocks	NR	CP patients (not disabled) with EPI	331
Fang K. 2022	Patients with benign or low-grade pancreatic tumors who have undergone pancreatectomy (6.9% of cohort have EPI)	Resource use	Length of hospital stay	NR	> Total<br>> Spleen-preserving distal pancreaticoduodenectomy<br>> Pylorus preserving pancreaticoduodenectomy<br>> Duodenum-preserving pancreatic head resection	> 101<br>> 25<br>> 7<br>> 7
Fang K. 2022	Patients with benign or low-grade pancreatic tumors who have undergone pancreatectomy (6.9% of cohort have EPI)	Resource use	Follow-up period	NR	> Total<br>> Spleen-preserving distal pancreaticoduodenectomy<br>> Pylorus preserving pancreaticoduodenectomy<br>> Duodenum-preserving pancreatic head resection	> 101<br>> 25<br>> 7<br>> 7

Author, year	Study population	Cost category	Cost description	Timepoint	Subgroup	Sample size
Gupta A. 2022	EPI patients	Direct costs	Average retail cost of five branded PERT therapies to treat EPI	NR	NA	NR
Gupta A. 2022	EPI patients	Direct costs	Cost of five branded PERT therapies to treat EPI when paid in coupons by patients	NR	NA	NR
Latenstein A.E.J. 2021	Patients with a history of pancreas surgery (64% of cohort have EPI)	Resource use	Length of hospital stay	Baseline	NA	153
Pranger B.K. 2021	PAYA patients who underwent pancreatic resection (27% of cohort have EPI)	Resource use	Length of hospital stay	NR	1. After pancreatoduodenectomy<br>2. After distal pancreatectomy	> 99<br>> 112
Pranger B.K. 2021	Adult patient ≥40 years who underwent pancreatic resection (27% of cohort have EPI)	Resource use	Length of hospital stay	NR	1. After pancreatoduodenectomy<br>2. After distal pancreatectomy	> 99<br>> 112
Pranger B.K. 2021	PAYA patients who underwent pancreatic resection (27% of cohort have EPI)	Resource use	Procedure time	NR	1. After pancreatoduodenectomy<br>2. After distal pancreatectomy	> 99<br>> 112
Bartholdy A. 2020	Patients with WON (22% of cohort have EPI)	Resource use	WON patients on PERT at discharge	NR	NR	125
Bartholdy A. 2020	Patients with WON (22% of cohort have EPI)	Resource use	Readmissions	NR	> WON-related<br>> non-WON-related	125
Dieguez-Castillo C. 2020	Patients with CP and EPI	Resource use	CP patients requiring PERT	NR	1. Patients with EPI<br>2. Patients with mild/moderate EPI<br>3. Patients with severe EPI	> 30<br>> 5<br>> 25

Author, year	Study population	Cost category	Cost description	Timepoint	Subgroup	Sample size
Dieguez-Castillo C. 2020	Patients with CP and EPI	Resource use	CP patients requiring surgical treatment	NR	1. Total with EPI<br>2. Patients with EPI on PERT<br>3. Patients with EPI w/o PERT<br>4. Patients with mild/moderate EPI<br>5. Patients with severe EPI	> 30<br>> 21<br>> 9<br>> 5<br>> 25
Eidt-Koch D. 2010	Patients with CF (82.4% of cohort have EPI)	Direct costs	Daily medication costs (Tobi®, Tobramycin)	2006	NA	NA
Eidt-Koch D. 2010	Patients with CF (82.4% of cohort have EPI)	Direct costs	Daily medication costs (Colistin®, Colistin)	2006	NA	NA
Eidt-Koch D. 2010	Patients with CF (82.4% of cohort have EPI)	Direct costs	Daily medication costs (Pulmozyme®, Dornase alfa)	2006	NA	NA
Eidt-Koch D. 2010	Patients with CF (82.4% of cohort have EPI)	Direct costs	Daily medication costs (Gernebcin®, Tobramycin)	2006	NA	NA
Eidt-Koch D. 2010	Patients with CF (82.4% of cohort have EPI)	Direct costs	Daily medication costs (Kreon 10000®, Pancreatin)	2006	NA	NA
Eidt-Koch D. 2010	Patients with CF (82.4% of cohort have EPI)	Direct costs	Daily medication costs (Kreon 25000®, Pancreatin)	2006	NA	NA
Eidt-Koch D. 2010	Patients with CF (82.4% of cohort have EPI)	Direct costs	Daily medication costs (Kreon 40000®, Pancreatin)	2006	NA	NA
Eidt-Koch D. 2010	Patients with CF (82.4% of cohort have EPI)	Direct costs	Daily medication costs (Sempera®, Itraconazole)	2006	NA	NA
Eidt-Koch D. 2010	Patients with CF (82.4% of cohort have EPI)	Direct costs	Daily medication costs (Zithromax®, Azithromycin)	2006	NA	NA
Eidt-Koch D. 2010	Patients with CF (82.4% of cohort have EPI)	Direct costs	Daily medication costs (Viani®, Fluticasone/salmeter)	2006	NA	NA
Eidt-Koch D. 2010	Patients with CF and EPI	Direct costs	Mean costs for CF patients with pancreatic insufficiency	2006	NA	NA

Author, year	Study population	Cost category	Cost description	Timepoint	Subgroup	Sample size
Eidt-Koch D. 2010	Patients with CF (0% patients had EPI, control group)	Direct costs	Mean costs for CF patients without pancreatic insufficiency	2006	NA	NA

CF: cystic fibrosis; CP: chronic pancreatitis; ED: emergency department; EPI: exocrine pancreatic insufficiency; IQR: interquartile range; NA: not applicable; NR: not reported; PAYA: pediatric, adolescent, and young adult; PERT: pancreatic enzyme replacement therapy; SD: standard deviation; USD: United States dollar; WON: walled-off necrosis
